# Clinical phenotype and outcomes in autoimmune encephalitis after herpes simplex virus encephalitis: A systematic review and meta-analysis

**DOI:** 10.1016/j.jinf.2025.106566

**Published:** 2025-08-06

**Authors:** Jonathan Cleaver, Renetta Chungath, Amy Gimson, Christine Strippel, Bryan Ceronie, Babak Soleimani, Thomas Johnson, Eyal Muscal, Kristen Fisher, Yike Jiang, Timothy A. Erickson, Kristy O. Murray, Siv Tonje Faret Hovet, Charlotte Aaberg Poulsen, Anna Søgaard Magnussen, Pauline Dumez, Shannon Ronca, Martin Häusler, Annegret Quade, Andrew Swayne, Morten Blaabjerg, Mette Nissen, Alexander J. Sandweiss, Jerome Honnorat, Russell Dale, Ming Lim, Michael Eyre, Margherita Nosadini, Lahiru Handunnetthi, Sarosh R. Irani, Adam E. Handel

**Affiliations:** a Oxford Autoimmune Neurology Group, Nuffield Department of Clinical Neurosciences, University of Oxford, Oxford, UK; b Department of Neurology, John Radcliffe Hospital, Oxford University Hospitals, Oxford, UK; c Department of Neurology, Southmead Hospital, Bristol, UK; d Wellcome Centre for Human Genetics, University of Oxford, Oxford, United Kingdom. Nuffield Department of Clinical Neurosciences, West Wing, John Radcliffe Hospital, University of Oxford, Oxford, UK; e Department of Pediatrics, Section of Neurology and Developmental Neuroscience, Baylor College of Medicine and Texas Children’s Hospital, Houston, TX, USA; f Department of Pediatrics, Section of Rheumatology, Baylor College of Medicine and Texas Children’s Hospital, Houston, TX, USA; g Department of Pediatrics, Division of Pediatric Rheumatology, Duke University School of Medicine, Durham, NC, USA; h Laboratories for Emerging and Tropical Disease Research, Department of Epidemiology & Biostatistics, Texas A&M School of Public Health, College Station, TX, USA; i Department of Pediatrics, Division of Innovation and Research, Emory University School of Medicine and Children’s Healthcare of Atlanta, Atlanta, GA, USA; j Department of Clinical Research, University of Southern Denmark, 5230 Odense, Denmark; k Department of Nuclear Medicine, Odense University Hospital, 5000 Odense, Denmark; l Department of Neurology, Odense University Hospital (OUH), Denmark; m Department of Neurosurgery, Copenhagen University Hospital - Rigshospitalet, Copenhagen, Denmark; n French Reference Centre on Paraneoplastic Neurological Syndromes and Autoimmune Encephalitis, Hospices Civils de Lyon, MeLiS- UCBL- CNRS UMR 5284. INSERM U1314, Université Claude Bernard Lyon 1, Lyon, France; o Department of Molecular Virology and Microbiology, Baylor College of Medicine, Houston, TX, USA; p Department of Pediatrics, Division of Tropical Medicine, Baylor College of Medicine, Houston, TX, USA; q Department of Pediatrics, Division of Neuropediatrics and Social Pediatrics, Medical Faculty, RWTH Aachen University, Aachen, Germany; r Mater Centre for Neurosciences, Mater Hospital, Brisbane, Queensland, Australia; s Brain and Mind Centre, Faculty of Medicine and Health, University of Sydney, Sydney, NSW, Australia; t Clinical Neuroimmunology Group, Kids Neuroscience Centre and Sydney Medical School, Faculty of Medicine and Health, University of Sydney, Sydney, NSW, Australia; u TY Nelson Department of Neurology, Children’s Hospital at Westmead, Sydney, NSW, Australia; v Children’s Neurosciences, Evelina London Children’s Hospital at Guy’s and St Thomas’ NHS Foundation Trust, London, UK; w Department of Women & Children’s Health, School of School of Life Course & Population Sciences, Faculty of Life Sciences and Medicine, King’s College London, London, UK; x Departments of Imaging Physics & Engineering and Early Life Imaging, School of Biomedical Engineering & Imaging Sciences, King’s College London, London, UK; y Paediatric Neurology and Neurophysiology Unit, Department of Women’s and Children’s Health, University Hospital of Padova, Padova, Italy; z Neuroimmunology Group, Paediatric Research Institute “Città della Speranza,” Padova, Italy; aa Department of Psychiatry, University of Oxford, Oxford, UK; ab Departments of Neurology and Neurosciences, Mayo Clinic, Jacksonville, FL, USA

**Keywords:** Herpes simplex virus encephalitis, Infectious encephalitis, Autoimmune encephalitis, NMDA receptor-antibody encephalitis, Neuroimmunology, Immunotherapy

## Abstract

**Background:**

Autoimmune encephalitis after herpes simplex virus encephalitis (HSVE-AE) represents the intersection of central nervous system infection and autoimmunity. Defining the phenotype and the safety and effectiveness of immunotherapy in HSVE-AE would help identify immunotherapy candidates, optimise therapeutic strategies, and improve patient outcomes.

**Methods:**

We systematically searched Embase, Medline, PubMed, and Web of Science (2007–2024) for cases meeting consensus criteria for AE after confirmed HSVE. Demographics, phenotype, treatment and outcome data were extracted. Dimensionality reduction, network analysis, and multivariate logistic regression was used to explore age- and diagnosis-specific patterns and outcome predictors.

**Results:**

From 2259 articles screened, 78 studies (225 patients) were included (median age 7.25 years; 52.9% female). Children (0–12 years) experienced more seizures during HSVE (p=0.003) and movement disorders during AE (p < 0.001). Older patients (> 12 years) had more headaches during HSVE (p=0.003), and speech dysfunction (p=0.02) and neuropsychiatric symptoms (p=0.02) during AE. HSVE-AE (89.3% N-methyl-D-aspartate receptor-antibody encephalitis [NMDAR-AbE]) differed significantly from a canonical NMDAR-AbE cohort (n=1550) in clinical, paraclinical and outcome domains.

Poor outcomes were linked to infant and older adult age, neuropsychiatric symptoms, and AE-phase mRS > 4. Rituximab independently predicted better outcomes. Disability improved over time (p < 0.001), with adverse event rates comparable to NMDAR-AbE.

**Conclusions and relevance:**

This meta-analysis defines novel age-specific HSVE-AE features, outcome predictors, and confirms the safety and improved outcomes of HSVE-AE after immunotherapy.

## Introduction

The global health burden of infection with herpes simplex virus (HSV) is high: there are an estimated 3.8 billion individuals under the age of 50 (64%) and 520 million aged 15–49 who have acquired HSV-1 and HSV-2, respectively.^1,2^ The World Health Organisation estimated 104,000 deaths were attributed to encephalitis in 2016, with herpes simplex virus (HSV) as the leading cause of infectious encephalitis in industrialised countries.^3^ HSV encephalitis (HSVE) results in inexorable central nervous system (CNS) inflammation with attendant significant neurological impairment. Since the introduction of acyclovir half a century ago, the mortality rate of HSVE has reduced substantially from 70% to 10%.^4^ Nevertheless, almost half of survivors exhibit moderate-to-severe disability, with fewer than 20% able to resume employment, underscoring the substantial socioeconomic burden of this disease.^5^

Building upon original clinical observations of choreoathetosis emerging months after HSVE,^6^ a retrospective study screened patients for the presence of N-methyl-D-aspartate receptor (NMDAR) antibodies and identified their presence in 30%.^7^ Subsequently, several groups reported patients with clinical features compatible with *bona fide* autoimmune encephalitis (AE) after HSVE (HSVE-AE), which is now thought to emerge in around 25% of cases.^8–11^ The emergence of pathogenic neuroglial surface autoantibodies (NSAbs) in HSVE-AE – most often targeting NMDAR – highlights a convergence between central nervous system (CNS) infection and autoimmunity.^7,12^ This recent observation has implications for tolerance breakdown and autoimmunisation against CNS antigens.

Importantly, it suggests that immunotherapy, rather than antiviral treatments, is beneficial in HSVE-AE. Indeed, reports from several cohorts have demonstrated improvements in outcomes following immunotherapy.^11,13–16^ Similarly, outcomes following the use of adjunct intravenous dexamethasone (10 mg QDS for 4 days) are being investigated in the DexEnceph multi-centre randomised control trial (ISRCTN11774734) for all cases of acute HSVE; a treatment which may provide protection against later development of AE. The trial has subsequently completed recruitment, and results are anticipated soon.

To assess the clinical spectrum and treatment in HSVE-AE, we conducted a systematic review and meta-analysis of all published cases to comprehensively profile its phenotypic complexity, outcome predictors, and the safety and efficacy of immunotherapy. Moreover, we used data derived from a previous meta-analysis assessing the outcomes and safety of immunotherapy following non-HSVE-associated NMDAR-antibody encephalitis (NMDAR-AbE) as a comparator cohort to identify clinically relevant differences.^17^ Through this comparative approach, we aimed to delineate clinically important differences between HSVE-AE and non-HSVE NMDAR-AbE, providing a detailed novel clinical profile, and guiding tailored HSVE-AE-specific therapeutic strategies to facilitate immunotherapy decisions.

## Methods

### Search strategy

A systematic review and meta-analysis were undertaken in accordance with the Preferred Reporting Items for Systematic Reviews and Meta-Analyses statement (PRISMA) guidelines. Searches for individual patient-level data were conducted in Ovid Embase, Medline, PubMed and Web of Science, using the terms ‘herpes simplex’, ‘encephalitis’, ‘HSVE’ and ‘HSE’ (see Supplementary Methods for further details). Reference lists and review articles were evaluated to find other relevant cohorts not identified in the databases. The search was not limited by age, gender, race or ethnicity. Studies published between January 1, 2007, and Sep 1, 2024, were included. Eligible studies involved confirmed HSVE defined by phenotypic compatibility and evidence of HSV-1 or HSV-2 in the cerebrospinal fluid (CSF) or brain tissue, confirmed by PCR or immunohistology confirmation. AE and NMDAR-AbE were defined using the Graus criteria in the presence of new or worsening of pre-existing CNS symptoms for longer than 24 h.^18^ Two reviewers (JC and RC) assessed cases with disagreements resolved by a third (AEH). Data extraction was conducted using a comprehensive data extraction form, which included demographics, phenotype, treatment, and outcomes during both HSVE and AE clinical phases. Authors from manuscripts reporting potential cases for inclusion were contacted, where missing data were identified to obtain the relevant raw dataset (Tables S9 & 10). Raw data from the landmark canonical NMDAR-AbE meta-analysis were provided by the corresponding authors for detailed univariate analyses.^17^ The review protocol was prospectively registered with PROSPERO (CRD42024522232).

### Outcomes of interest

The primary outcome was the modified Rankin Scale (mRS) score, with poor outcome defined as an mRS of > 2. The mRS was modified for the paediatric cohort, in accordance with an adapted version from Bigi et al.^19^ Secondary outcomes included neuropsychiatric sequelae, seizures, anti-seizure medication use, HSV recurrence defined by the reemergence in the CSF and immunotherapy-associated adverse events.

### Statistical analysis

**Fig. 1:**
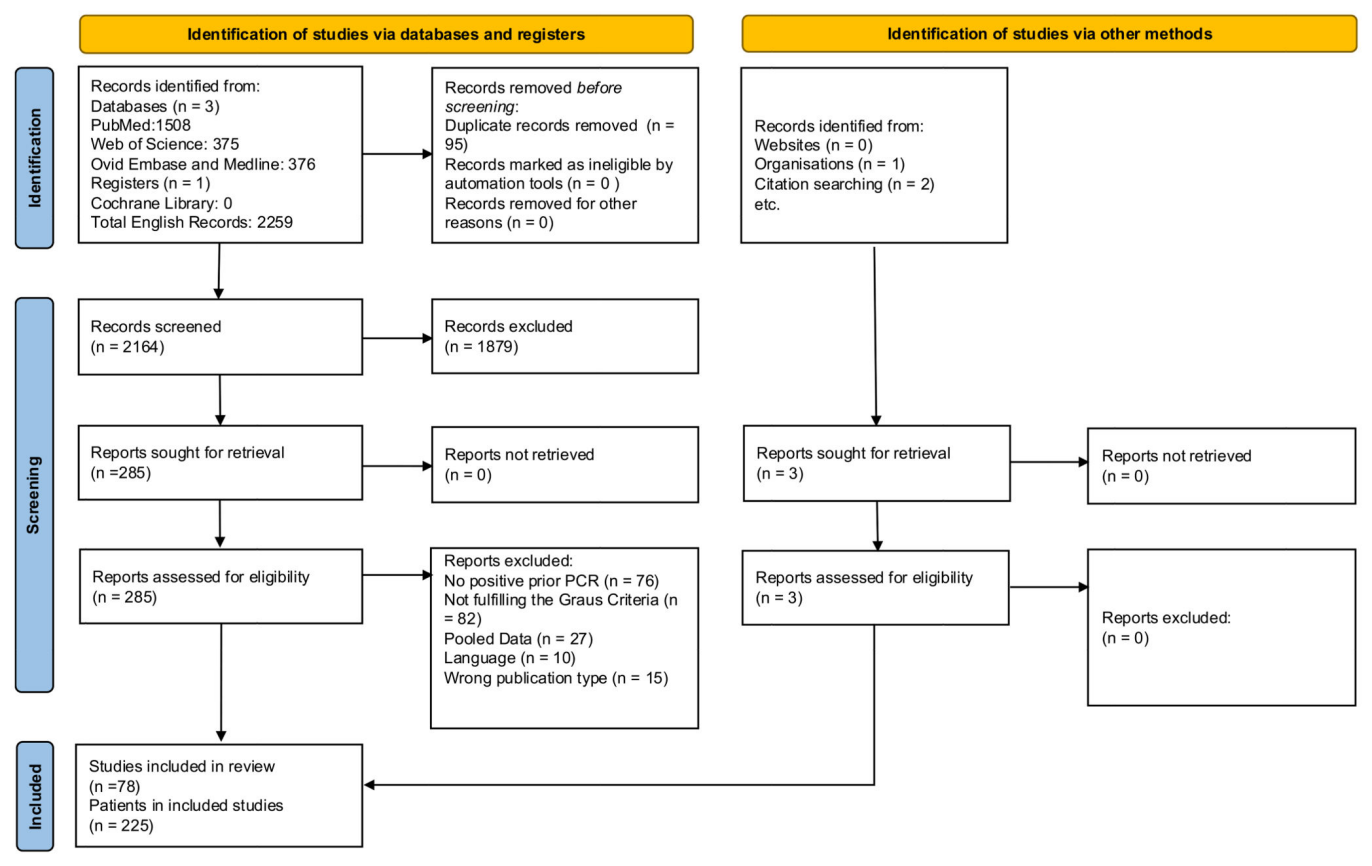
PRISMA diagram.

To analyse the spread of complex phenotypic data in two-dimensional space, dimensionality reduction was performed using ‘FactoMineR’ and ‘Factoextra’, including all variables reported in ≥80% of cases. Initial age group clustering was guided by the spread of the data dictated through the ‘quantile’ function in base R (Fig. 1A). Detailed inter-relationship analysis of the clinical and paraclinical features was presented in a network plot using ‘igraph’. Similar to previous methods,^20^ variables with > 10% the frequency of the maximally observed feature were included. We compared symptom network properties between age groups using the ‘NetworkComparr’ package, applying a non-parametric permutation-based test (10,000 iterations) to assess group differences in centrality (closeness, betweenness, strength, and expected influence).

Multivariate logistic regression was used to predict outcomes (favourable: mRS 0–2 vs. poor mRS > 2). To prepare the data, except for outcomes which were unimputed, a 10% K-nearest neighbour imputation method was used and predictor variables with > 10% missing data were excluded leaving 15 individual predictor variables and reducing the final set of observations to 154. Backward stepwise regression using the Akaike Information Criterion was employed and multicollinearity was assessed using a Variance Inflation Factor. A 20–80 test-and-train split was used to evaluate the performance of the model. Odds ratios and 95% confidence intervals were extracted from the coefficients of the final model.

Sensitivity analyses were undertaken to address the impact of missing data and the inclusion of Armangue et al.^11^ which included non-random data missingness (supplementary figure 3). A risk of bias assessment was performed using the JBI critical appraisal tool (supplementary tables 9 and 10).^21^

Chi-squared and Mann-Whitney U tests were used for categorical and continuous variables, respectively. Benjamini-Hochberg corrections were applied for tests with multiple comparisons to reduce the probability of type I errors. Spearman’s rank correlation coefficients were calculated for correlative analyses. Statistically significant values were defined as p-values < 0.05 and denotated as < 0.05*, < 0.01** and < 0.001***. Analyses were undertaken in R (version 2024.09.0+375) and Microsoft Excel (version 16.90.2).

### Role of the funding source

The funder of the study had no role in study design, data collection, data analysis, data interpretation, or writing of the report. The corresponding author had full access to all the data in the study. All authors had final responsibility for the decision to submit for publication.

## Results

From 2259 screened articles across 4 databases, we included data from 78 studies (225 patients) (Fig. 1; Tables S1-10). The median age was 7.25 years (IQR 43 years) with 52.9% (117/221) females. The median time from HSVE diagnosis to AE onset was 28 days (IQR 23 days). There were associated tumours identified in 3 (3.7%, [3/81]) patients: 2 involving the nervous system – paraclinoid meningioma and optic nerve tumour – and the third with thymic hyperplasia.

During their index HSVE illness, most patients (52.3%, [57/109]) had extensive supratentorial inflammation on MRI with 3 or more brain lobar involvement. CSF often showed a pleocytosis (90.9%, [97/103]) with elevated protein (66.7%, [97/103]) and red blood cell counts (66.7%, [26/39]). During the AE illness, electroencephalogram (EEG) was frequently abnormal (86.8% [79/91]) with predominant focal or diffuse slow-wave activity. CSF pleocytosis was found in 90% (117/130) with elevated protein in 52% (65/125), but few oligoclonal bands (19.4%, [21/108]). HSV was detected in 16.6% (32/193) of CSF samples during the AE phase. Additional MRI-brain T2 and/or FLAIR hyperintensities were identified in 91.5% (118/129) with frequent reports of new contrast enhancement (44/76, [57.8%]).

Most patients became positive for NMDAR antibodies (89.3%, [201/225]) with 22.6% (31/137) demonstrating evidence of other CNS autoantibodies alone or in combination with NMDAR antibodies. Other autoantibodies included: contactin-associated protein-like 2 (CASPR2), leucine-rich glioma-inactivated 1 (LGI1), glutamic acid decarboxylase (GAD), myelin oligodendrocyte glycoprotein (MOG), gamma-aminobutyric acid A receptor (GABA_A_R), GABA_B_R, alpha-amino-3-hydroxy-5-methyl-4-isoxazolepropionic acid receptors (AMPAR), glial fibrillary acidic protein (GFAP), metabotropic gluta-mate receptor 5 (mGluR5), and dopamine 2 receptor, as well as antibodies directed against unknown antigenic targets.

Most patients received immunotherapy (90.7%) with a median time to treatment from AE onset of 9 days (IQR 24 days) and a median time from AE onset to last follow-up of 12 months (IQR 18 months). First-line immunotherapy was administered in 90.2% (203/225), which included: corticosteroids (80.9%, [182/225]); intravenous immunoglobulins (63.1%, [142/225]); plasma exchange (76%, [171/225]). Second-line immunotherapy was administered in 45.8% ([103/225]) of patients: rituximab (RTX) (42.2%, [95/225]) and cyclophosphamide (13.8%, [31/225]). There was no significant difference in time to first- and second-line immunotherapy during the AE illness between HSV CSF PCR positive and negative cohorts (p=0.92).

Other medical treatments administered during HSVE-AE included anti-seizure medications (61.5%, [80/130]), acyclovir (57.4%, [81/141]), antipsychotics (29.5%, [33/112]), and medications to ameliorate movement disorders (13.5%, [15/111]) and dysautonomias (7.1%, [7/98].

**Fig. 2:**
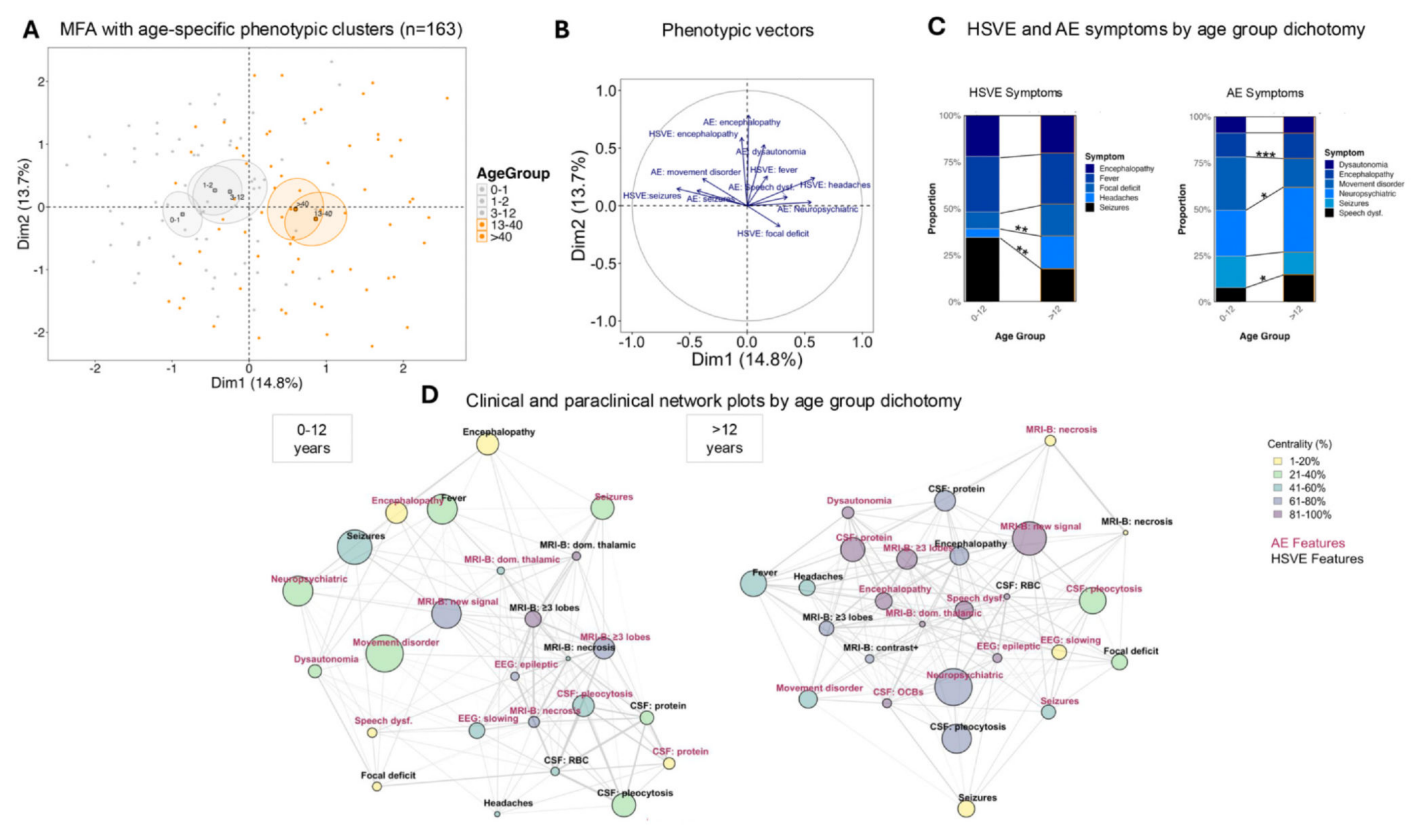
Phenotypic heterogeneity of HSVE and HSVE-AE. A) Multiple factor analysis clustered by age reveals a dichotomy in HSVE and HSVE-AE phenotypic heterogeneity emerging at the age of 12. Age-specific clusters were guided by quantiles supplying an equal split of the dataset. B) Specific symptoms are hierarchically ranked by the contribution to the dimension reductions and presented in vector-weighted contributions. C) Stacked bar charts split by the MFA-dictated age dichotomy reveal significant symptom-specific differences. p-values shown are adjusted for multiple comparisons. D) Network analysis of the clinical and paraclinical features of HSVE and HSVE-AE. The clinical and paraclinical features are represented as nodes; the size of the nodes is proportionate to the frequency of the feature and the edge thickness is proportionate to the frequency of co-occurrences. The nodes are colour-coded by closeness centrality, a measure of interconnectedness. CSF = cerebrospinal fluid; EEG = electroencephalogram; FLAIR = fluid-attenuated inversion recovery; OCB = oligoclonal band; RBC = red blood cells.

Dimensionality reduction of all clinical features into two dimensions identified maximal phenotypic heterogeneity between 0–12 and > 12 years (Fig. 2A-B; Fig. S1). In HSVE, seizures were more frequently observed in the younger cohort (χ^2^ =10.8; p = 0.003) whereas more headaches were observed > 12 years (χ^2^ = 11.8; p = 0.003) (Fig. 1C). Within the younger 0–12-year cohort, AE presented more frequently with movement disorders (χ^2^=14.2; p < 0.001) whereas > 12-year-olds experienced more neuropsychiatric symptoms (χ^2^ = 7.1; p = 0.05) and speech dysfunction (χ^2^ = 6.6; p = 0.02) (Fig. 2C). NMDAR antibodies were more prevalent in the 0–12-year-old group (χ^2^ = 10.2; p = 0.003) and other NSAbs were most prominent > 12-years-old (χ^2^ = 9.14; p = 0.005).

Age-specific network structures identified unique age group-specific clusters interlinking multiple clinical and paraclinical factors (Fig. 2D). In the 0–12-year cohort, HSVE-associated fever and seizures, and AE-associated MRI-brain abnormalities (new signal and ≥3 lobe involvement) demonstrate high frequency and centrality. Raised CSF red blood cell count in HSVE interconnected strongly with brain necrosis during AE; dominant thalamic involvement in HSVE with seizures in AE; and ≥3 lobe involvement in HSVE were linked with necrotic brain changes and epileptiform EEG abnormalities during AE. In the > 12-year cohort, HSVE phases were characterised by central features of encephalopathy and CSF pleocytosis and raised protein. During the AE phase, neuropsychiatric symptoms, speech dysfunction, raised CSF protein, and new MRI-brain signal were most central. In contrast to the 0–12-year cohort, interconnectedness between HSVE and AE phases in this older cohort was less pronounced. Overall, relevant HSVE and HSVE-AE clinical symptoms are shared amongst all age groups. However, the centrality of the HSVE-AE clinical and paraclinical networks is clearly distinct (Table S11).

To assess for HSVE-AE-specific features (n=225; 89.3%, [201/225] NMDAR-AbE), we compared this cohort to a non-HSV-associated NMDAR-AbE cohort^17^ (n=1550) (Table 1, S1–4 and 6). We found that HSVE-AE patients were typically younger (median age 7.25 versus 20 years; p < 0.001) with a less marked sex ratio (52.9% female versus 73.3%; χ^2^ =38.5; p < 0.001). HSVE-AE patients appeared to experience fewer severe presentations: seizures (40.9% versus 68.3%; χ^2^ =62.3; p < 0.001), decreased consciousness (36% versus 55.4%; χ^2^ = 28.6; p < 0.001) and autonomic dysregulation (24.7% versus 43.2%; χ^2^ =26.7; p < 0.001) were all significantly lower than the non-HSVE-associated NMDAR-AbE cohort.

CSF was more frequently cellular (80.3% versus 67.8%; χ^2^ =8.2; p=0.01) with elevated protein (52% versus 21.6%; χ^2^ =53; p < 0.001) in HSVE-AE but CSF-restricted oligoclonal bands were more frequent in NMDAR-AbE (62.6% versus 19.4%; χ^2^ =62.0; p < 0.001). EEG abnormalities were comparable between both cohorts, with focal or diffuse slow waves being the predominant features and extreme delta brush observed in 6% (5/84). Fewer associated neoplasms were identified in HSVE-AE patients (3.7% versus 25.6%; χ^2^ =19.8; p < 0.001).

Immunotherapy paradigms were similar except for more frequent use of plasma exchange (PLEX) in traditional NMDAR-antibody encephalitis (33.7% versus 24% in HSVE-AE; χ^2^ =8.45; p=0.01). Unexpectedly, a higher proportion of patients were administered immunotherapy within 30 days (78.7% versus 59.8%; χ^2^ =38.5 p < 0.001) in the HSVE-AE cohort. There were no differences in immunotherapy commencement between HSV CSF PCR positive versus negative patients (Table S5). Rituximab (RTX) was used more commonly and promptly in HSVE-AE (42.6% versus 24.5%; χ^2^ = 33; p < 0.001; with 62% versus 13.1% given within 30 days; χ^2^ = 45.2; p < 0.001). Overall, outcomes were worse with HSVE-AE than canonical NMDAR-AbE (median mRS 3 versus 2; p < 0.001) but with lower mortality (1.4% versus 6.3%; χ^2^ = 8.71; p=0.009).

**Fig. 3:**
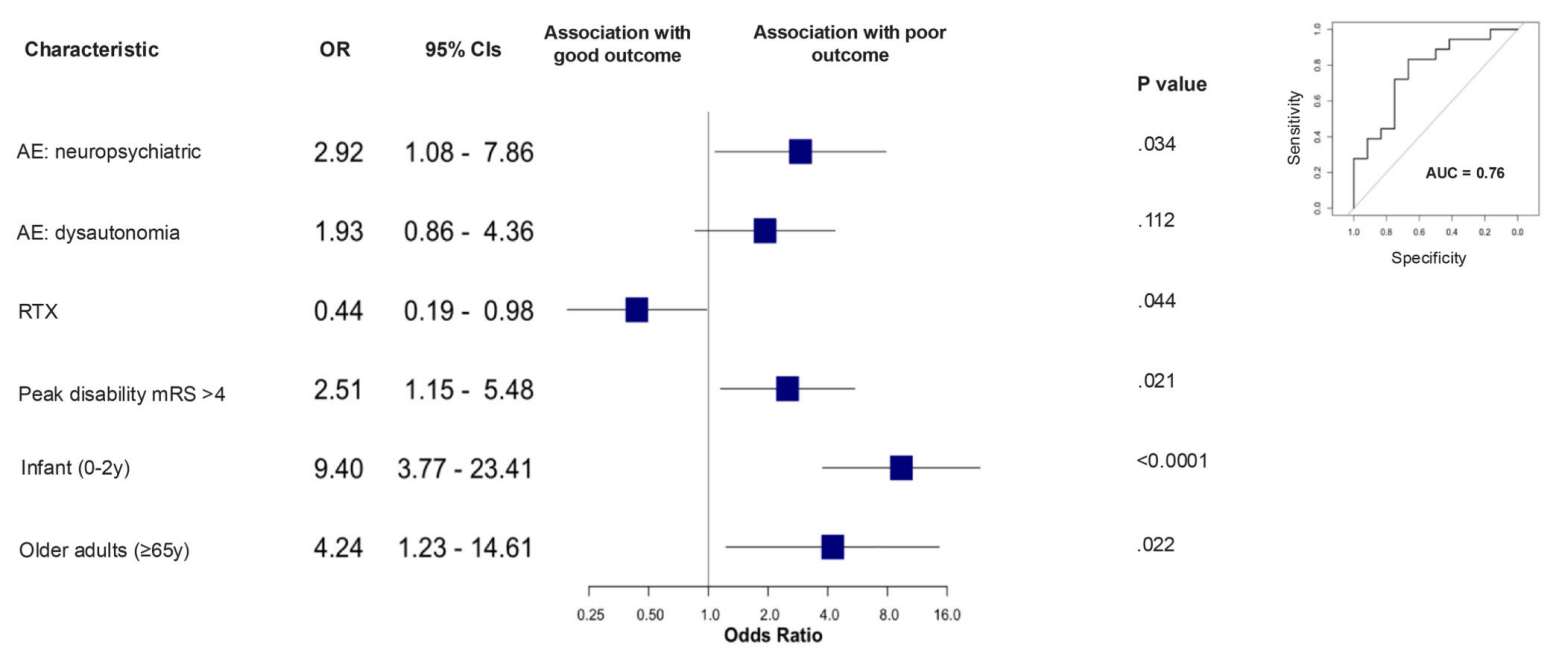
Multiple logistic regression model for HSVE-AE outcomes. Results from a backward stepwise logistic regression model excluding Armangue et al.^11^ and with 10% KNN imputation are displayed. Reports with 10% or more missing data were also excluded leaving n=154 in the final model. Outcomes are defined as good by an mRS 0–2 and poor by mRS > 2. AE = autoimmune encephalitis; mRS = modified Rankin scale; RTX = rituximab.

We included 154 patients in the final regression model to predict overall outcomes defined as favourable (mRS 0–2) or poor (mRS > 2) (Fig. 3; Fig. S2). Demographic predictors of follow-up mRS-defined poor outcomes paralleled findings from traditional NMDAR-AbE including infancy (0–2 years) (OR 9.4, 95% CI: 3.77–23.41; p < 0.001) and age > 65 years (OR 4.24, 95% CI: 1.23–14.61; p=0.02). Clinical features associated with poor outcomes included neuropsychiatric symptoms in the HSVE-AE phase (OR 2.92, 95% CI: 1.08–7.86; p=0.03) and peak disability mRS > 4 at HSVE-AE disease nadir (OR 2.51, 95% CI: 1.15–5.48; p=0.02). The only factor associated with improved outcomes was use of the B-cell depleting medication, RTX (OR 0.44, 95% CI: 0.19–0.98; p=0.04; Fig. S2A-C). The model performed with a 73% accuracy and an area under the curve (AUC) of 0.76. Results from the other regression models, including this dataset without imputation and the full dataset, are shown in Fig. S2.

**Fig. 4:**
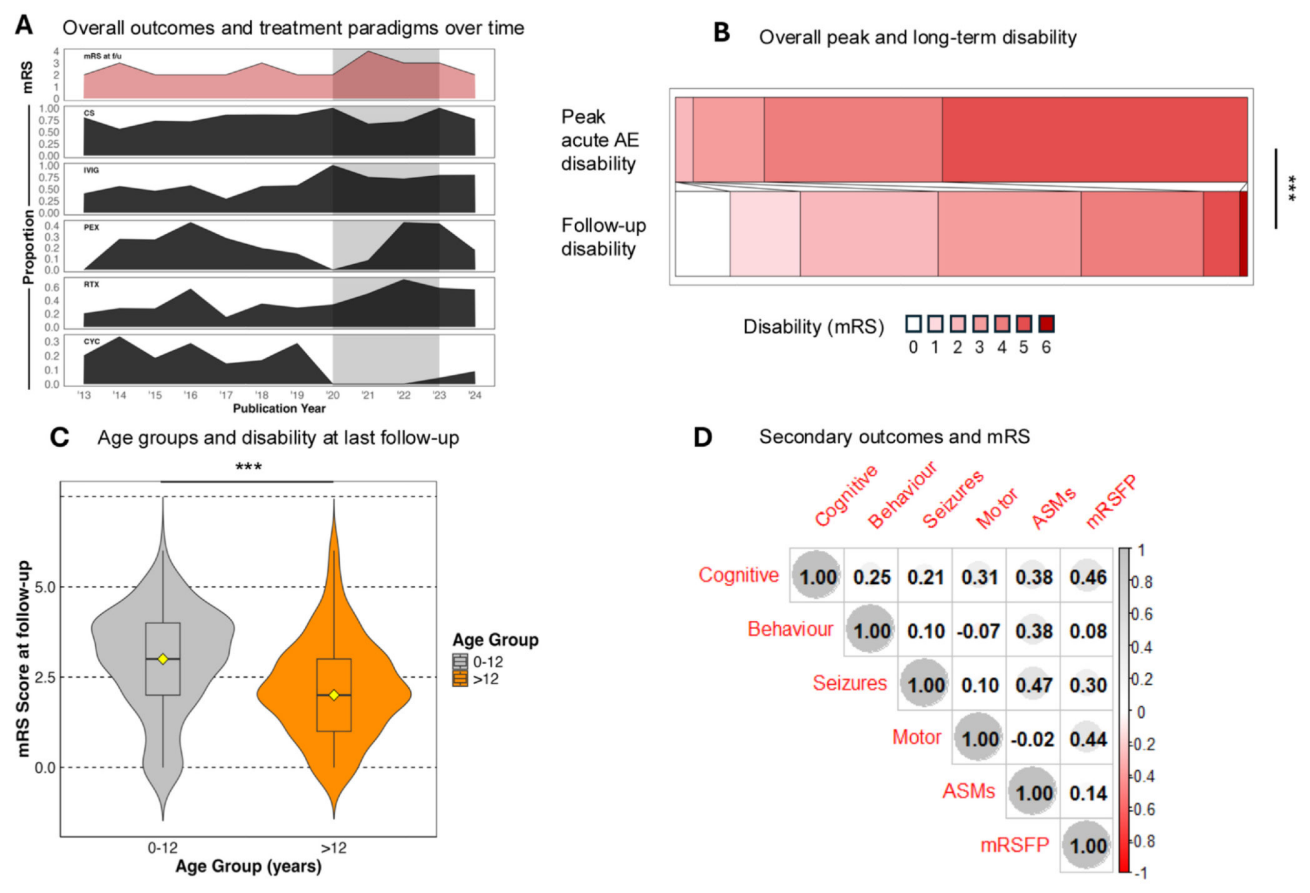
Treatment and outcomes of HSVE-AE. A) Immunosuppression treatment and follow-up disability over time by publication year revealing changes in second-line immunotherapy with similar outcomes. Grey shaded area highlighting the COVID-19 pandemic epoch. B) Peak acute AE phase disability is plotted in parallel alongside follow-up disability showing significant improvements across the overall cohort. C) mRS defined disability outcomes at follow-up, divided into 0–12- and > 12-year age groups. D) Correlogram displaying relationships across all primary and secondary outcomes. Positive correlations are depicted in grey and negative correlations in red. The size of the circle is proportional to the strength of the correlation with the R value depicted in each circle. Behaviour and neuropsychiatric manifestations appear under the label “behaviour”. ASMs = anti-seizure medications; CS – corticosteroids; CYC = cyclophosphamide; IVIG = intravenous immunoglobulins; PEX = plasma exchange; RTX = rituximab; mRSFP = mRS at follow-up.

We further assessed treatment and outcome trends across the entire cohort. Treatment paradigms varied over time from the first confirmed publications in 2013, with more pronounced fluctuations focussing around the COVID-19 pandemic epoch. Strikingly, cyclophosphamide was administered to patients less frequently, whereas RTX usage increased across the COVID-19 pandemic. PLEX usage also reduced in frequency around the pandemic, whereas steroid and IVIg administration remained broadly the same. Despite evolving immunotherapy strategies, mRS-defined outcomes remained relatively stable (Fig. 4A).

Overall, patients proportionately improved from a median peak admission AE mRS of 5 (IQR 1) to 3 at last follow-up (IQR 2; χ^2^ = 183.4 p < 0.001; Fig. 4B). There were age-dependent differences in outcomes (Fig. 4C): follow-up mRS was higher in 0–12-year-olds than those > 12-year-old (χ^2^ = 19.9, p < 0.001; Fig. 4C) with this cohort experiencing increased rates of epilepsy (χ^2^ =11.4; p=0.002 and motor deficits (χ^2^ =24.4; p < 0.001). Cognitive (74.3%, [153/206]) and motor problems (32.1%, [65/203]) were the principal drivers of long-term disability (Fig. 4D). In contrast to canonical NMDAR-AbE, while significantly poorer outcomes did correlate independently with longer time-to-treatment (p=0.04; Fig. S3A&B), this did not reach significance in multivariate regression analysis (OR 1.77, CI: 0.44–7.1; Fig. S3C).

Immunotherapy-associated adverse events were comparable to canonical NMDAR-AbE (3.3% versus 3.8%, respectively; χ^2^ =0.1; p=0.8), which included hospital acquired infections, vascular events, haemodynamic instability secondary to PLEX and one case of diabetes insipidus (Tables S1, S8). One patient treated with steroids died from bowel ischaemia due to thrombosis of the mesenteric artery.

## Discussion

This comprehensive systematic review and meta-analysis makes several observations about the recently described phenomena of HSVE-AE.^11^ We found that a 12-year age cut-off was a key determinant of phenotypic heterogeneity, highlighted key clinical and paraclinical differences compared to traditional NMDAR-AbE, identified features associated with poor outcomes (extremes of ages [0–2, and > 65 years], peak disability and neuropsychiatric symptoms) or good outcomes (RTX), and demonstrated that immunotherapy was associated with few adverse events.

The emergence of maximal phenotypic heterogeneity at 12 years represents a watershed for age-dependent clinical trajectories in HSVE-AE, signifying junctures in disease progression and response to treatment. For instance, seizures and movement disorders are more frequent in the younger cohort, whereas neurobehavioural, headache and speech dysfunction symptoms are more salient clinical hallmarks in those > 12 years. This finding substantiates a smaller cohort study reviewing differences between adults and teenagers with younger children.^22^ Importantly, many clinical features frequently observed in the > 12-year cohort, assumed to be naturally higher than the younger cohort, were not solely responsible for maintaining this phenotypic dichotomy. Moreover, additional network structures incorporating paraclinical data demonstrated age-specific differences of additional clinical utility: new MRI-brain necrosis during AE central to 0–12-year group; AE-phase encephalopathy, speech dysfunction, raised CSF protein, epileptiform abnormalities on EEG central to > 12-year group; and severe brain inflammation affecting ≥3 brain lobes and new T2/FLAIR hyperintensities central to both cohorts.

Further phenotypic granularity is provided through comparison with a large dataset of non-HSVE-associated NMDAR-AbE. This broadly reveals a less fulminant NMDAR-AbE presentation in HSVE-associated disease and a lower associated mortality. However, disability was higher in HSVE-AE, although it was challenging to determine the contribution of HSVE or AE to the final outcomes. Neoplasms were rarely identified in patients with HSVE-AE; however, notably, two of the three affected individuals had neoplasms involving the nervous system. It could be speculated that local malignancies could facilitate a dysregulated local inflammatory milieu, predisposing certain individuals to acquisition of HSVE and/or later AE – an observation requiring continued vigilance and analysis in larger future cohorts. Finally, time-to-immunotherapy was expedited in the HSVE-AE cohort, which could reflect heightened suspicion for possible development of AE following confirmed pre-disposing HSVE.

Poor outcomes in the 0–2-year and > 65 year subgroups reflect similar findings in canonical NMDAR-AbE.^17^ This observation in the younger cohort may reflect a more severe immune response to primary HSV infection – which is more common to this age group – than latent reactivation.^23^ Moreover, inherited immune dysfunctions are found frequently in infancy, especially inborn errors involving the toll-like receptor (TLR)-dependent interferon response pathways.^24–27^ Patients > 65-years-old also have worse outcomes, potentially due to lack of cognitive reserve or frailty. Moreover, immune senescence in older patients is known to impair host control of the certain infections.^28^ In turn, this could allow more severe HSV-mediated brain inflammation and senescence-related pro-inflammatory states could, in part, predispose to later CNS autoimmunity; the latter synonymous with other age-related autoimmune diseases.^29^

HSV was detected in the CSF of 16.6% AE patients, predominantly affecting younger patients with a median age of 7.25 years. Notwithstanding, time-to-immunotherapy was not affected in comparison to the HSV CNS negative cohort (Supplementary Table 5). This suggests immunotherapy does not significantly contribute to poorer outcomes in patients, previously treated with a sufficient course of acyclovir, in the AE illness phase. Overall, treatment with acyclovir prior to CSF testing is recommended and there is no evidence to delay immunotherapy if AE is confirmed.

This persistent viral detection within the CNS raises further hypotheses about this patient cohort. This observation could suggest ongoing viral-mediated neural damage and host CNS epitope release ultimately serves as a substrate for peripheral autoimmunisation. Indeed, a recent study supports the association between ongoing neuroaxonal damage – assessed by measuring serum neurofilament light chain levels – with subsequent AE.^30^ However, the fact that NMDAR-AbE is not frequently observed after other forms of brain injury suggests this is not sufficient for AE and that additional, yet unknown viral-specific and host factors are also necessary for CNS autoimmunity.

Overall, the eventual level of disability improved from the admission AE-phase mRS nadir. First-line immunotherapies were administered in the majority (90.7%, [201/225]), making it challenging to identify any of these as significant predictors of outcomes. However, RTX use was associated with improved outcomes. Together with the observation of a low frequency of CSF-restricted OCBs, this indirectly suggests that targeting B cells involved in peripheral germinal centre-based autoantibody production process is beneficial, as seen in other forms of NSAb-mediated diseases.^31,32^ Importantly, although an essential prognostic factor in canonical NMDAR-AbE, time-to-immunotherapy did not correlate significantly with overall outcomes for HSVE-AE. However, the timing of immunotherapy was insufficiently described in many reports, which highlights the importance of future studies to detail this information.

This study represents the most comprehensive systematic review and meta-analysis of patients with HSVE-AE to-date, incorporating a rigorous framework, systematic quality assessment, and detailed subgroup analysis. However, there were several limitations. The main limitations of this study include the reporting heterogeneity and retrospective nature of case reports which are subject to biases including reporting patients with atypical presentations. There is potential bias of reporting specific symptoms by age but sensitivity analysis suggested that this was not a key driver of age-specific heterogeneity (Fig. S2B&C). Moreover, the reports often lacked detailed data on the disability accrued after index HSVE, making it difficult to attribute any improvement specifically to immunotherapy. The rare nature of HSVE-AE necessarily means that the number of cases available for analysis was limited. However, our findings were consistent across multiple iterations of regression and clustering analysis. Finally, case reports and cohorts were geographically biased to Europe, reinforcing the need for broader global datasets for future analyses.

## Conclusion

This study identifies significant phenotypic variations in viral and autoimmune encephalitis across distinct age groups, offering crucial insights for tailored diagnostic and therapeutic strategies. By high-lighting clinically actionable predictors of outcomes and providing robust evidence for the safety and effectiveness of immunotherapy in HSVE-AE, these findings have the potential to substantially influence clinical management guidelines and therapeutic protocols. There remains a critical need for comprehensive and standardised reporting of phenotypic data within prospective cohorts of HSVE across diverse populations. Addressing this gap will enhance the generalisability of clinical predictions and outcomes in HSVE-AE, ultimately driving improvements in patient care and informing healthcare treatment decisions.

## Supplementary Material

Supplementary data associated with this article can be found in the online version at doi:10.1016/j.jinf.2025.106566.

Supplementary Material

## Figures and Tables

**Table 1:** Significant clinical differences between HSVE-AE and a canonical NMDAR-AbE cohort.

Domain		HSVE-AE cohort^a^ N= 225	Nosadini et al. (2021) N=1550	*P*-value^b^
Demographics	Female	52.9%	73.3%	< 0.001
	Age at Onset	Median: 7.25;	Median: 20; Mean: 22.97; IQR: 16.3	< 0.001
		Mean: 21.70;		
		IQR: 43		
Clinical	Seizures	40.9% (91/222)	68.3% (944/1382)	< 0.001
	Decreased level of consciousness	36.0% (80/222)	55.4% (744/1343)	< 0.001
	Autonomic dysfunction	24.7% (55/222)	43.2% (583/1351)	< 0.001
Paraclinical	Abnormal MRI	98.5% (140/142)	40.6% (434/1069)	< 0.001
	CSF: pleocytosis	79.5% (102/127)	67.8% (618/911)	0.01
	CSF: elevated proteins	52.0% (65/125)	21.6% (189/873)	< 0.001
	CSF: oligoclonal bands	19.4% (21/108)	62.6% (223/356)	< 0.001
	Tumour presence	3.7% (3/81)	25.6% (389/1524)	< 0.001
Treatment	Acyclovir use	57.4% (81/141)	19.8% (220/1113)	0.004
	Anti-seizure medication use	61.5% (80/130)	40.8% (461/1129)	0.004
	Plasma exchange use	24% (54/225)	33.7% (500/1482)	0.01
	Second-line immunotherapy	46.2% (103/225)	31.8% (486/1526)	0.004
	Rituximab	42.6% (96/225)	24.5% (363/1484)	0.004
	Long-term Rituximab	3.7% (8/214)	0.5% (7/1508)	0.004
	Time from AE to immunotherapy ≤30 days	78.7% (100/127)	59.8% (277/463)	< 0.001
	Time between onset and Rituximab ≤30	62.0% (31/50)	13.1% (18/137)	< 0.001
Outcome	Good Outcome at Follow-up (mRS ≤2)	45.8% (104/225)	71.5% (1108/1550)	< 0.001

a HSVE-AE cohort included 225 patients with 89.3%, [201/225] NMDAR-AbE.

b Chi-squared tests with Benjamini-Hochberg post-hoc corrections for categorical and Mann-Whitney U tests were used for continuous data.

## Data Availability

All data used for this study have been included in the manuscript and supplementary material.

## Panel: Research in Context

### Evidence before this study

The World Health Organisation estimated 104,000 deaths were attributed to encephalitis in 2016, with HSV as the leading cause of infectious encephalitis in industrialised countries. HSVE is traditionally thought to generate significant and often irreversible morbidity with no active treatment paradigms beyond anti-viral therapy. Clinicians are becoming increasingly aware of the recently described phenomena of AE following HSVE, although deep phenotypic data, clinically useful predictors for outcomes and the effectiveness and safety of immunotherapy strategies are limited. We systematically searched Ovid Embase, Medline, PubMed, and Web of Science from Jan 1, 2007, to Sep 1, 2024, for studies on autoimmune encephalitis (AE) or NMDAR-antibody encephalitis following confirmed herpes simplex virus encephalitis (HSVE). Inclusion criteria were clearly defined cases meeting consensus diagnostic criteria. Studies not meeting these criteria or lacking sufficient clinical data were excluded. The search terms included combinations of “autoimmune encephalitis,” “herpes simplex virus encephalitis,” “NMDAR-antibody encephalitis,” and related synonyms. The evidence quality was variable, given the observational nature and heterogeneity of included studies. No systematic reviews or meta-analyses of this kind have been published to date.

### Added value of this study

This individual patient meta-analysis consolidates previously fragmented evidence, clarifies age-specific clinical phenotypes and highlights critical predictors of clinical outcomes in HSVE-AE. By using robust statistical methods, including multiple factor analyses, network analyses, and multivariate logistic regression, we demonstrated significant differences compared to canonical NMDAR-antibody encephalitis, identified rituximab as an independent predictor of improved outcomes, and confirmed immunotherapy’s safety profile.

### Implications of all the available evidence

Our findings have significant clinical implications, emphasising the necessity for age-tailored assessment and management strategies in HSVE-AE. The identification of predictive factors such as age, neuropsychiatric symptoms, and disability severity at AE-phase nadir can guide clinicians in prognosis and treatment decisions. The confirmed safety and effectiveness of immunotherapy, particularly rituximab, support its broader clinical implementation, particularly in the context of a previous infectious encephalitis. To reduce the significant HSVE-related health burden, future research should focus on prospective studies and HSVE clinical trials to further validate these findings.

## References

[1] Looker KJ, Magaret AS, May MT, Turner KME, Vickerman P (2015). Global and regional estimates of prevalent and incident herpes simplex virus type 1 infections in 2012. PLoS One.

[2] Looker KJ, Magaret AS, Turner KME, Vickerman P, Gottlieb SL, Newman LM (2015). Global estimates of prevalent and incident herpes simplex virus type 2 infections in 2012. PLoS One.

[3] World Health Organization EC for DC (2018). Global health estimates 2016: deaths by cause, age, sex, by country and by region, 2000–2016.

[4] McGrath N, Anderson NE, Croxson MC, Powell KF (1997). Herpes simplex encephalitis treated with acyclovir: diagnosis and long term outcome. J Neurol Neurosurg Psychiatry.

[5] Harris L, Griem J, Gummery A, Marsh L, Defres S, Bhojak M (2020). Neuropsychological and psychiatric outcomes in encephalitis: a multi-centre case-control study. PLoS One.

[6] De Tiège X, Rozenberg F, Des Portes V, Lobut JB, Lebon P, Ponsot G (2003). Herpes simplex encephalitis relapses in children: differentiation of two neurologic entities. Neurology.

[7] Prüss H, Finke C, Höltje M, Hofmann J, Klingbeil C, Probst C (2012). N-methyl-D-as-partate receptor antibodies in herpes simplex encephalitis. Ann Neurol.

[8] Mohammad SS, Sinclair K, Pillai S, Merheb V, Aumann TD, Gill D (2014). Herpes simplex encephalitis relapse with chorea is associated with autoantibodies to N-Methyl-D-aspartate receptor or dopamine-2 receptor. Mov Disord.

[9] Hacohen Y, Deiva K, Pettingill P, Waters P, Siddiqui A, Chretien P (2014). N-methyl-D-aspartate receptor antibodies in post-herpes simplex virus encephalitis neurological relapse. Mov Disord.

[10] Armangue T, Leypoldt F, Málaga I, Raspall-Chaure M, Marti I, Nichter C (2014). Herpes simplex virus encephalitis is a trigger of brain autoimmunity. Ann Neurol.

[11] Armangue T, Spatola M, Vlagea A, Mattozzi S, Cárceles-Cordon M, Martinez-Heras E (2018). Frequency, symptoms, risk factors, and outcomes of autoimmune encephalitis after herpes simplex encephalitis: a prospective observational study and retrospective analysis. Lancet Neurol.

[12] Cleaver J, Jeffery K, Klenerman P, Lim M, Handunnetthi L, Irani SR (2023). The immunobiology of herpes simplex virus encephalitis and post-viral autoimmunity. Brain.

[13] Dumez P, Villagrán-García M, Bani-Sadr A, Benaiteau M, Peter E, Farina A (2024). Specific clinical and radiological characteristics of anti-NMDA receptor autoimmune encephalitis following herpes encephalitis. J Neurol.

[14] Quade A, Rostasy K, Wickström R, Aydin ÖF, Sartori S, Nosadini M (2023). Autoimmune encephalitis with autoantibodies to NMDAR1 following herpes encephalitis in children and adolescents. Neuropediatrics.

[15] Søgaard A, Poulsen CA, Belhouche NZ, Thybo A, Hovet STF, Larsen L (1953). Post-herpetic anti-NMDAR encephalitis in Denmark: current status and future challenges. Biomedicines.

[16] Sandweiss AJ, Erickson TA, Jiang Y, Kannan V, Yarimi JM, Fisher K (2023). Infectious profiles in pediatric anti-N-methyl-d-aspartate receptor encephalitis. J Neuroimmunol.

[17] Nosadini M, Eyre M, Molteni E, Thomas T, Irani SR, Dalmau J (2021). Use and safety of immunotherapeutic management of N-methyl-d-aspartate receptor antibody encephalitis: a meta-analysis. JAMA Neurol.

[18] Graus F, Titulaer MJ, Balu R, Benseler S, Bien CG, Cellucci T (2016). A clinical approach to diagnosis of autoimmune encephalitis. Lancet Neurol.

[19] Bigi S, Fischer U, Wehrli E, Mattle HP, Boltshauser E, Bürki S (2011). Acute ischemic stroke in children versus young adults. Ann Neurol.

[20] Al-Diwani A, Handel A, Townsend L, Pollak T, Leite MI, Harrison PJ (2019). The psycho-pathology of NMDAR-antibody encephalitis in adults: a systematic review and phenotypic analysis of individual patient data. Lancet Psychiatry.

[21] Munn Z, Barker TH, Moola S, Tufanaru C, Stern C, McArthur A (2020). Methodological quality of case series studies: an introduction to the JBI critical appraisal tool. JBI Evid Synth.

[22] Armangue T, Moris G, Cantarín-Extremera V, Conde CE, Rostasy K, Erro ME (2015). Autoimmune post-herpes simplex encephalitis of adults and teenagers. Neurology.

[23] Abel L, Plancoulaine S, Jouanguy E, Zhang SY, Mahfoufi N, Nicolas N (2010). Age-dependent Mendelian predisposition to herpes simplex virus type 1 encephalitis in childhood. J Pediatr.

[24] Zhang SY (2020). Herpes simplex virus encephalitis of childhood: inborn errors of central nervous system cell-intrinsic immunity. Hum Genet.

[25] Zhang SY, Casanova JL (2024). Genetic defects of brain immunity in childhood herpes simplex encephalitis. Nature.

[26] Zhang SY, Jouanguy E, Ugolini S, Smahi A, Elain G, Romero P (2007). TLR3 deficiency in patients with herpes simplex encephalitis. Science.

[27] Casrouge A, Zhang SY, Eidenschenk C, Jouanguy E, Puel A, Yang K (2006). Herpes simplex virus encephalitis in human UNC-93B deficiency. Science.

[28] Simon AK, Hollander GA, McMichael A (2015). Evolution of the immune system in humans from infancy to old age. Proc R Soc B Biol Sci.

[29] Liu Z, Liang Q, Ren Y, Guo C, Ge X, Wang L (2023). Immunosenescence: molecular mechanisms and diseases. Signal Transduct Target Ther.

[30] Falk KK, Cabrera LA, Junker R, Leypoldt F, Malter MP, Markewitz R (2025). Serum NfL predicts outcome and secondary autoimmunity in herpes-simplex encephalitis. J Neuroimmunol.

[31] Damato V, Theorell J, Al-Diwani A, Kienzler AK, Makuch M, Sun B (2022). Rituximab abrogates aquaporin-4-specific germinal center activity in patients with neuromyelitis optica spectrum disorders. Proc Natl Acad Sci USA.

[32] Al-Diwani A, Theorell J, Damato V, Bull J, McGlashan N, Green E (2022). Cervical lymph nodes and ovarian teratomas as germinal centres in NMDA receptor-antibody encephalitis. Brain.

