# Supplementary material for: Clinical phenotype and outcomes in autoimmune encephalitis after herpes simplex virus encephalitis: A systematic review and meta-analysis
Source: J Infect. Author manuscript; Available in PMC 2026 Jan 29. (PMC7618681; doi:10.1016/j.jinf.2025.106566)
Supplement: Supplementary Material [file EMS212239-supplement-Supplementary_Material.zip › 1-s2.0-S0163445325001604-mmc1.docx]

**Supplementary methods**

*Search strategy*

A systematic review and meta-analysis were undertaken in accordance with the Preferred reporting items for systematic reviews and meta-analyses statement (PRISMA) guidelines. The search was conducted for case and cohort studies in patients with post-herpes simplex virus autoimmune encephalitis (HSVE-AE). The search was not limited by age, gender, race or ethnicity. HSVE required clinical diagnoses, through evidence of HSV-1 or HSV-2 in the CSF or brain tissue, confirmed by PCR or immunohistology confirmation. Secondary autoimmune encephalitis was defined as the exhibition of new or worsening of pre-existing central nervous system system for longer than 24 hours and in accordance with the Graus Criteria.^1^

The search was conducted in PubMed, Ovid Embase and Medline, Web of Science and Cochrane reviews, using the keywords ‘herpes simplex’ OR ‘herpes simplex encephalitis’ AND ‘autoimmune encephalitis’. Each search was restricted to English language publications after 2007, as this is when the NMDA receptor antibody was first described. Reference lists and review articles were evaluated to find other relevant cohorts not identified in the databases.

| **Databases and Registers** | **Search Strategy** |
| --- | --- |
| **PubMed** | "herpes encephalitis"[All Fields] OR "encephalitis, herpes simplex"[MeSH Terms] OR "herpes simplex"[MeSH Terms] OR "HSE"[Text Word] OR "HSVE"[Text Word] OR “herpes simplex”[Text Word] OR “herpes simplex encephalitis”[Text Word] AND "autoimmune diseases of the nervous system"[MeSH Terms] OR "anti n methyl d aspartate receptor encephalitis"[MeSH Terms] OR "anti n methyl d aspartate receptor encephalitis"[MeSH Terms] OR "n methylaspartate"[MeSH Terms] OR "autoantibodies"[Text Word] OR "autoimmune encephalitis"[Text Word] OR "NMDA"[Text Word] OR "encephalitis"[MeSH Terms] |
| **Ovid Embase and Medline** | Herpes simplex encephalitis.  Broader terms   - herpetic encephalitis   Narrower terms   - Herpes simplex 1/2 encephalitis - Herpes simplex meningoencephalitis   AND  Autoimmune encephalitis   - Narrower terms - Anti NMDA receptor encephalitis - Autoimmune limbic encephalitis - Autoimmune meningoencephalitis - Hashimoto encephalitis |
| **Web of Science** | (("herpes encephalitis") OR ("herpes simplex encephalitis") OR ("herpes simplex virus  encephalitis") OR ("HSE") OR ("HSVE"))  AND  ("Autoimmune encephalitis") OR ("autoantibody encephalitis") OR ("antibody-mediated  encephalitis") OR ("autoantibody-mediated encephalitis") OR (“limbic encephalitis”) OR  ("NMDA") OR ("antibody") OR ("N-methyl-D-aspartate") |

From the search, case and cohort studies with a confirmed HSVE diagnosis, a presentation of AE, a mention of immunotherapy and clinical outcomes were included while other studies were excluded. Authors from manuscript were contacted where missing data was identified to obtain the relevant raw dataset (see supplementary tables 9 and 10).

Data extraction was conducted using a comprehensive data extraction form, which included age, sex, clinical and paraclinical data for HSVE and AE, timings, treatments and outcomes. The primary outcome measure was the modified Rankin Scale (mRS) score at follow up, modified for the paediatric cohort, in accordance with an adapted version of Bigi et al.^2^ The scale was adapted to start at 0 instead of 1; this allowed it to be directly aligned with the adult modified Rankin scale, which was used in the larger scale study.

*Statistical analysis*

Dimensionality reduction was performed using ‘FactoMineR’ and ‘factoextra’. Variables with >20% missing data were excluded. Initial age group clustering was guided by the spread of the data dictated through the ‘quantile’ function in base R (figure 1A). A dichotomy in grouping emerged and subsequent iterations were employed to identify maximal cluster separation (supplementary figure 2A).

**Supplementary table 1** Demographics, clinical and paraclinical features

|  | **Total Cohort** | **Adult** | **Paediatric (<18 years)** | ***p value***  ***(adult vs. paediatric; padj^a^ in brackets)*** | **Nosadini et al 2021**^6^ | ***p value***  ***(Overall: HSVE-AE vs. Nosadini et al, padj in brackets)*** |
| --- | --- | --- | --- | --- | --- | --- |
| **Demographics** | | | | | | |
| *Total number of patients* | 225 | 89 | 136 |  | 1550 |  |
| *Female* | 117/221  (52.9%) | 48/88 (54.5%) | 65/133  (48.9%) | 0.41 (0.68) | 1105/1508 (73.3%) | <0.001 |
| *Age at onset* | Median: 7.25  Mean: 21.70  Range: 0-84 (83.94) | Median: 50  Mean: 49.5  Range: 18-84 (66) | Median: 1.313  Mean: 3.51  Range: 0-17 (16.94) |  | Median: 20, mean: 22.97, range: 0-85 (1517/1550) | <0.001 |
| **Clinical characteristics - HSE** | | | | | | |
| *Encephalopathy* | 76/140  (54.3%) | 28/55  (50.9%) | 48/85  (56.5%) | 0.52 (0.68) | N/A | N/A |
| *Focal neurological deficit* | 45/140  (32.14%) | 29/55  (52.7%) | 19/85  (22.4%) | <0.001 (0.002) | N/A | N/A |
| *Fever* | 103/140  (73.6%) | 39/55  (70.9%) | 64/85  (75.3%) | 0.57 (0.68) | N/A | N/A |
| *Seizures* | 96/140  (68.6%) | 25/55  (45.5%) | 71/85  (83.5%) | <0.001 (<0.001) | N/A | N/A |
| *Headaches* | 38/140  (27.1%) | 22/55  (40.0%) | 19/85  (22.4%) | 0.03 (0.08) | N/A | N/A |
|  | **Total Cohort** | **Adult** | **Paediatric (<18 years)** | ***p value*** | **Nosadini et al 2021** | ***p value*** |
| **Investigations - HSE** | | | | | | |
| *MRI-B: ≥3 lobes involved* | 57/109  (52.3%) | 19/54  (37.0%) | 38/55  (69.0%) | <0.001 (<0.001) | N/A | N/A |
| *MRI-B: contrast enhancement* | 24/94  (25.5%) | 15/50  (30.0%) | 9/44  (20.5%) | 0.29 (0.47) | N/A | N/A |
| *MRI-B: necrosis* | 17/94  (18.1%) | 7/46  (15.2%) | 10/48  (20.8%) | 0.48 (0.68) | N/A | N/A |
| *MRI-B: dominant thalamic hyperintensity* | 22/106  (20.8%) | 4/51  (7.8%) | 18/55  (32.7%) | 0.002 (0.008) | N/A | N/A |
| *CSF: pleocytosis* | 97/103  (90.9%) | 46/49  (93.9%) | 51/54  (94.4%) | 0.9 (0.9) | N/A | N/A |
| *CSF: RBC raised* | 26/39  (66.7%) | 9/16  (56.3%) | 17/23  (73.9%) | 0.25 (0.43) | N/A | N/A |
| *CSF: elevated proteins* | 63/87  (72.4%) | 35/44  (79.5%) | 28/43  (65.1%) | 0.13 (0.3) | N/A | N/A |
| *CSF: HSV PCR or nMGS* | 223/224^b^  (99.6%) | 87/88  (98.8%) | 136/136  (100%) | N/A | 0/1550 | N/A |
| **Clinical Characteristics - AE** | | | | | | |
| *Abnormal (psychiatric) behaviour or cognitive dysfunction* | 177/222  (79.7%) | 79/88  (89.8%) | 98/134  (73.1%) | 0.003 (0.01) | 1177/1409 (83.5%) | 0.16 (0.25) |
| *Speech dysfunction (pressured speech, verbal reduction, mutism)* | 65/218  (29.8%) | 36/88  (40.9%) | 29/130  (22.3%) | 0.003 (0.01) | 521/1341 (38.9%) | 0.01 (0.02) |
| *Seizures* | 91/222  (40.9%) | 20/88  (22.7%) | 61/134  (45.5%) | <0.001 (0.003) | 944/1382 (68.3%) | <0.001 (<0.001) |
|  | **Total Cohort** | **Adult** | **Paediatric (<18 years)** | ***p value*** | **Nosadini et al 2021** | ***p value*** |
| *Movement disorder* | 140/223  (62.7%) | 35/88  (39.8%) | 105/135  (77.8%) | <0.001 (<0.001) | 855/1357 (63%) | 0.95 (0.95) |
| *Decreased level of consciousness* | 80/222  (36.0%) | 33/88  (37.5%) | 47/134  (35.1%) | 0.71 (0.8) | 744/1343 (55.4%) | <0.001 (<0.001) |
| *Autonomic dysfunction or central hypoventilation* | 55/222  (24.7%) | 21/88  (23.9%) | 34/134  (25.4%) | 0.8 (0.9) | 583/1351 (43.2%) | <0.001 (<0.001) |
| *Other symptoms: sleep-wake cycle disturbances* | 52/222  (23.4%) | 24/88  (27.3%) | 24/134  (17.9%) | 0.01 (0.24) | 223/1359 (16.4%) | 0.01 (0.02) |
| *Other symptoms: flu-like prodromal symptoms <1 month earlier* | 26/222  (11.7%) | 9/88  (10.2%) | 17/134  (12.7%) | 0.58 (0.68) | 233/1361 (17.1%) | 0.04 (0.07) |
| *Headache at presentation* | 15/218  (6.9%) | 7/84  (8.3%) | 8/134  (6.0%) | 0.51 (0.68) | N/A | N/A |
| **Investigations - AE** |  |  |  |  |  |  |
| *EVER Abnormal EEG (focal or diffuse slow or disorganised activity, epileptic activity, or extreme delta brush)* | 79/91  (86.8%) | 39/44  (88.6%) | 40/47  (85.1%) | 0.62 (0.71) | 725/855 (84.8%) | 0.6 (0.7) |
| *Focal or diffuse slow or disorganised activity* | 56/84  (66.7%) | 26/40  (65%) | 30/44  (68.1%) | 0.76 (0.83) | 594/822 (72.3%) | 0.28 (0.37) |
| *Epileptic activity (epileptic discharges)* | 33/85  (38.8%) | 16/41  (39%) | 17/44  (38.6%) | 0.97 (0.97) | 244/829 (29.4%) | 0.07 (0.12) |
| *Seizures recorded?* | 13/84  (15.5%) | 5/40  (12.5%) | 8/44  (18.2%) | 0.47 (0.68) | 132/829 (15.9%) | 0.92 (0.95) |
|  | **Total Cohort** | **Adult** | **Paediatric (<18 years)** | ***p value*** | **Nosadini et al 2021** | ***p value*** |
| *EEG status epilepticus recorded?* | 2/84  (2.4%) | 2/40  (5.0%) | 0/44  (0.0%) | 0.13 (0.3) | 43/827 (5.2%) | 0.26 (0.36) |
| *Extreme delta brush* | 5/84  (6%) | 1/40  (2.5%) | 4/44  (9.1%) | 0.2 (0.4) | 56/827 (6.8%) | 0.77 (0.85) |
| *EEG PLEDs* | 2/84  (2.4%) | 0/40  (0%) | 2/44  (4.5%) | 0.17 (0.37) | 13/827 (1.6%) | 0.58 (0.69) |
| *EVER CSF with pleocytosis or oligoclonal bands* | 117/130  (90.0%) | 59/62  (95.2%) | 58/68  (85.3%) | 0.06 (0.17) | 692/924 (74.9%) | <0.001 (<0.001) |
| *CSF: pleocytosis* | 102/127  (80.3%) | 52/63  (82.5%) | 50/64  (78.1%) | 0.53 (0.68) | 618/911 (67.8%) | 0.005 (0.01) |
| *RBC* | 10/55  (18.2%) | 5/23  (21.7%) | 5/32  (15.6%) | 0.56 (0.68) | N/A | N/A |
| *CSF: elevated protein* | 65/125  (52.0%) | 40/64  (62.5%) | 25/61  (41.0%) | 0.02 (0.06) | 189/873 (21.6%) | <0.001 (<0.001) |
| *CSF: oligoclonal bands* | 21/108  (19.4%) | 16/56  (28.6%) | 5/52  (9.6%) | 0.013 (0.05) | 223/356 (62.6%) | <0.001 (<0.001) |
| *CSF PCR for HSV positive* | 32/193  (16.6%) | 11/84  (13.1%) | 21/107  (19.6%) | 0.23 (0.42) | N/A | N/A |
| *Abnormal Brain MRI* | 140/142  (98.5%) | 60/60  (100%) | 80/82  (97.6%) | 0.22 (0.42) | 434/1069 (40.6%) | <0.001 (<0.001) |
| *T2 and FLAIR hyperintensities* | 118/129  (91.5%) | 54/58  (93.1%) | 64/71  (90.1%) | 0.55 (0.68) | N/A | N/A |
|  | **Total Cohort** | **Adult** | **Paediatric (<18 years)** | ***p value*** | **Nosadini et al 2021** | ***p value*** |
| *Dominant thalamic T2 and FLAIR hyperintensities* | 25/125  (20.0%) | 9/57  (15.8%) | 16/68  (23.5%) | 0.28 (0.47) | N/A | N/A |
| *MRI ≥3 lobes involved* | 77/127  (60.6%) | 30/58  (51.7%) | 47/69  (68.1%) | 0.06 (0.17) | N/A | N/A |
| *MRI: necrosis* | 42/114  (36.8%) | 17/51  (33.3%) | 25/63  (39.7%) | 0.48 (0.68) | N/A | N/A |
| *Contrast enhancement* | 44/76  (57.8%) | 24/41  (58.5%) | 20/35  (57.1%) | 0.9 (0.92) | N/A | N/A |
| *Brain biopsy* | 2/225  (0.9%) | 2/89  (2.2%) | 0/136  (0.0%) | 0.08 (0.21) | N/A | N/A |
| *Serum NMDAR antibodies* | 164/192  (85.4%) | 57/78  (73.1%) | 107/114  (93.9%) | <0.001 (<0.001) | 702/796 (88.2%) | 0.29 (0.37) |
| *CSF NMDAR antibodies* | 120/159  (75.5%) | 37/65  (56.9%) | 83/94  (88.3%) | <0.001 (<0.001) | 973/986 (98.7%) | <0.001 (<0.001) |
| *Other neuroglial surface antibodies* | 31/137  (22.6%) | 23/60  (38.3%) | 8/77  (10.4%) | <0.001 (<0.001) | N/A | N/A |
| *Tumour* | 3/81  (3.7%) | 3/34  (8.8%) | 0/47  (0.0%) | 0.04 (0.12) | 389/1524 (25.6%) | <0.001 (<0.001) |

PCR = polymerase chain reaction; PLEDS = periodic lateralised epileptiform discharges

^a^Adjusted p values (including those in the tables hereafter) were corrected by applying the Benjamini-Hochberg method.

^b^Patient had a positive brain biopsy on post-mortem.

**Supplementary table 2** Tumour associations

| **Patient ID** | **Tumour** | **Treatment** | **mRS at follow-up** |
| --- | --- | --- | --- |
| *103* | Tumour compressing right optic nerve. | Pterional craniotomy prior to HSVE onset | 1 |
| *132* | Paraclinoid meningoma | Surgical removal | 3 |
| *152* | Thymic hyperplasia | Thymectomy | 0 |

**Supplementary table 3** Treatments

| **Treatment - HSE** | **Total Cohort** | **Adult** | **Paediatric** | ***p value***  **(*padj)*** | **Nosadini et al 2021 (n=1550)** | ***p value***  **(*padj)*** |
| --- | --- | --- | --- | --- | --- | --- |
| *Acyclovir* | 140/140  (100%) | 59/59  (100%) | 81/81  (100%) | N/A | N/A | N/A |
| *Symptomatic treatments* | 49/54  (90.7%) | 30/33  (90.9%) | 19/21  (90.5%) | 0.96 | N/A | N/A |
| **Treatment - AE** |  |  |  |  |  |  |
| *Anti-seizure medications* | 80/130  (61.5%) | 35/58  (60.3%) | 45/72  (58.3%) | 0.8 (0.91) | 461/1129  (40.8%) | <0.001 (<0.001) |
| *Acyclovir* | 81/141  (57.4%) | 36/57  (63.2%) | 45/84  (53.6%) | 0.26 (0.55) | 220/1113  (19.8%) | <0.001 (<0.001) |
| *Antipsychotics* | 33/112  (29.5%) | 20/49  (40.8%) | 13/63  (20.6%) | 0.02 (0.06) | 234/1119  (20.9%) | 0.04 (0.08) |
| *Treatment for movement disorder* | 15/111  (13.5%) | 2/48  (4.2%) | 13/63  (20.6%) | 0.01 (0.06) | 234/1119  (20.9%) | 0.06 (0.12) |
| *Treatment for dysautonomias* | 7/98  (7.1%) | 4/41  (10.0%) | 3/57  (5.3%) | 0.39 (0.68) | 22/1065  (1.4%) | 0.002 (0.006) |
| **Treatment - HSE** | **Total Cohort** | **Adult** | **Paediatric** | ***p value***  **(*padj)*** | **Nosadini et al 2021 (n=1550)** | ***p value***  **(*padj)*** |
| *First-line immunotherapy* | 204/225  (90.7%) | 79/89  (88.7%) | 125/136  (91.9%) | 0.39 (0.68) | 1395/1528 (91.3%) | 0.6 (0.8) |
| *Corticosteroids* | 183/225  (81.3%) | 69/89  (77.5%) | 114/136  (83.8%) | 0.24 (0.55) | 1205/1485 (81.1%) | 0.95 (0.95) |
| *Intravenous immunoglobulin* | 143/225  (63.5%) | 48/89  (53.9%) | 95/136  (69.9%) | 0.02 (0.06) | 980/1476 (66.4%) | 0.4 (0.56) |
| *Plasma exchange/ Therapeutic apheresis* | 54/225  (24.0%) | 14/89  (15.7%) | 40/136  (29.4%) | 0.02 (0.06) | 500/1482 (33.7%) | 0.004 (0.01) |
|  |  |  |  |  |  |  |
| *Second-line immunotherapy* | 103/225  (45.7%) | 28/89  (34.5%) | 72/136  (52.9%) | 0.002 (0.01) | 486/1526 (31.8%) | <0.001 (<0.001) |
| *Rituximab* | 96/225  (42.6%) | 25/89  (28.1%) | 71/136  (52.2%) | <0.001 (0.007) | 363/1484 (24.5%) | <0.001 (<0.001) |
| *Cyclophosphamide* | 31/225  (13.8%) | 11/89  (13.6%) | 20/136  (14.7%) | 0.62 (0.84) | 184/1484 (24.5%) | 0.56 (0.72) |
| *Long-term immune suppression?* | 28/214  (13.1%) | 12/89 (13.4%) | 16/125  (12.8%) | 0.88 (0.93) | 146/1508 (9.7%) | 0.12 (0.22) |
| *Long-term Intravenous immunoglobulin* | 6/214  (2.8%) | 2/81  (2.5%) | 4/114  (3.5%) | 0.68 (0.86) | 24/1508 (1.6%) | 0.2 (0.33) |
| *Mycophenolate mofetil* | 7/214  (3.3%) | 1/81  (1.2%) | 5/114  (4.3%) | 0.21 (0.55) | 43/1508 (2.9%) | 0.73 (0.8) |
| *Long-term Rituximab* | 8/214  (3.7%) | 3/81  (3.7%) | 5/114  (4.3%) | 0.81 (0.91) | 7/1508 (0.5%) | <0.001 (<0.001) |
| *Long-term steroids* | 8/214  (3.7%) | 4/81  (4.9%) | 4/114  (3.5%) | 0.62 (0.84) | 40/1508 (2.7%) | 0.37 (0.55) |
| **Treatment - HSE** | **Total Cohort** | **Adult** | **Paediatric** | ***p value***  **(*padj)*** | **Nosadini et al 2021 (n=1550)** | ***p value***  **(*padj)*** |
| *Adverse Events to Immunotherapy* | 7/212^a^  (3.3%) | 4/83  (4.8%) | 3/129  (2.3%) | 0.32 (0.61) | 52/1386  (3.8%) | 0.75 (0.8) |

^a^3/7 were hospital acquired infections, while the remaining 4 were directly linked to immunotherapy

**Supplementary table 4** Autoimmune encephalitis conversion and immunosuppression timing

| ***Time To AE (days)*** | **Total Cohort** | **Adult** | **Paediatric** | ***P (padj)*** | **Nosadini et al 2021 (n=1550** | ***P (padj)*** |
| --- | --- | --- | --- | --- | --- | --- |
| Time: HSVE-AE ≤20 | 46/195  (23.6%) | 17/71  (24.0%) | 13/67  (19.4%) | 0.52 (0.52) | N/A | N/A |
| Time: HSVE-AE ≤30 | 114/195  (58.5%) | 35/71  (49.3%) | 48/67  (71.6%) | 0.007 (0.08) | N/A | N/A |
| Time: HSVE-AE ≤60 | 167/195  (85.6%) | 56/71  (78.9%) | 61/67  (91.0%) | 0.05 (0.26) | N/A | N/A |
| Time: HSVE-AE ≤90 | 178/195  (91.3%) | 62/71  (87.3%) | 64/67  (95.5%) | 0.47 (0.51) | N/A | N/A |
| **Time To Treatment (days)** | **Total Cohort** | **Adult** | **Paediatric** | ***p value (padj)*** | **Nosadini et al 2021 (n=1550** | ***p value (padj)*** |
| *Time: AE-IT ≤20* | 88/127  (69.3%) | 33/53  (62.2%) | 55/74  (74.3%) | 0.15 (0.32) | 196/463  (42.3%) | <0.001 (<0.001) |
| *Time: AE-IT ≤30* | 100/127  (78.7%) | 39/53  (73.6%) | 61/74  (82.4%) | 0.23 (043) | 277/463  (59.8%) | <0.001 (<0.001) |
| *Time: AE-IT ≤60* | 113/129  (87.6%) | 45/55  (81.8%) | 68/74  (91.9%) | 0.09 (0.3) | 385/463  (83.2%) | 0.22 (0.26) |
| **Time To Treatment (days)** | **Total Cohort** | **Adult** | **Paediatric** | ***p value (padj)*** | **Nosadini et al 2021 (n=1550** | ***p value (padj)*** |
| *Time: AE-IT ≤90* | 116/129  (89.9%) | 47/55  (85.5%) | 69/74  (93.2%) | 0.15 (0.32) | 410/463  (88.6%) | 0.66 (0.66) |
| *Time:AE-2IT ≤30* | 32/52  (61.5%) | 5/6  (83.3%) | 27/41  (65.9%) | 0.39 (0.48) | 22/1101  (2.0%) | <0.001 (<0.001) |
| *Time:AE-2IT ≤60* | 43/52  (82.7%) | 8/11  (72.7%) | 35/41  (85.4%) | 0.33 (0.48) | 62/101  (61.4%) | 0.02 (0.03) |
| *Time:AE-2IT ≤120* | 45/51  (88.2%) | 8/10  (80.0%) | 37/41  (90.2%) | 0.37 (0.48) | 28/101  (27.7%) | <0.001 (<0.001) |
| *Time: AE-RTX ≤30* | 31/50  (62.0%) | 4/10  (44.4%) | 27/40  (67.5%) | 0.11 (0.3) | 18/137  (13.1%) | <0.001 (<0.001) |
| *Time: AE-RTX ≤60* | 41/50  (82.0%) | 7/10  (77.8%) | 34/40  (85.0%) | 0.27 (0.48) | 71/121  (58.7%) | 0.004 (0.006) |

IT = immunotherapy; RTX = rituximab

**Supplementary table 5** Immunosuppression timing and CSF HSV PCR positivity during HSVE-AE

| **Time To Treatment (days)** | **HSV+ Cohort** | **HSV- Cohort** | ***p value (padj)*** |
| --- | --- | --- | --- |
| *Time: AE-IT ≤20* | 10/14  (71.4%) | 77/106  (72.6%) | 0.92 (0.92) |
| *Time: AE-IT ≤30* | 11/14  (78.6%) | 87/106  (82.1%) | 0.75 (0.92) |
| *Time: AE-IT ≤60* | 12/14  (85.7%) | 93/106  (87.7%) | 0.83 (0.92) |
| *Time: AE-IT ≤90* | 13/14  (92.9%) | 95/106  (89.6%) | 0.7 (0.92) |
| *Time: AE-2IT ≤30* | 8/11  (72.7%) | 23/37  (62.2%) | 0.52 (0.92) |
| *Time: AE-2IT ≤60* | 10/11  (90.1%) | 31/37  (83.8%) | 0.56 (0.92) |
| *Time: AE-2IT ≤120* | 10/11  (90.1%) | 33/37  (89.2%) | 0.87 (0.92) |

**Supplementary table 6** Disability outcomes

|  | **Total cohort** | **Adult** | **Paediatric** | ***p value***  ***(padj)*** | **Nosadini et al 2021 (n=1550)** | ***p value (padj)*** |
| --- | --- | --- | --- | --- | --- | --- |
| **Overall median peak mRS in acute phase** | 5 (range 2-5) | 4 (range 2-5) | 5 (range 2-5) | 0.03 (0.05) | 5 (range 2-5) | 0.032 (0.05) |
| **Overall mRS at follow-up** | 3 (range 0-6) | 2 (range 0-6) | 3 (range 0-6) | 0.006 (0.03) | 2 (range 0-6) | <0.001 (<0.001) |
|  | **Total cohort** | **Adult** | **Paediatric** | ***p value (padj)*** | **Nosadini et al 2021 (n=1550)** | ***p value (padj)*** |
| **Worst mRS in Acute Phase** |  |  |  |  |  |  |
| *5* | 120/225  (53.3%) | 39/89  (43.8%) | 81/136  (59.6%) | 0.02 (0.06) | 652/1113 (58.6%) | 0.15 (0.23) |
| *4* | 70/225  (31%) | 35/89  (39.3%) | 35/136  (25.7%) | 0.03 (0.06) | 309/1113 (27.8%) | 0.31 (0.41) |
| *3* | 27/225  (12.0%) | 15/89  (18.9%) | 13/136  (9.6%) | 0.11 (0.17) | 144/1113 (12.9%) | 0.7 (0.8) |
| *2* | 7/225  (3.1%) | 0/89  (0.0%) | 7/136  (5.2%) | 0.03 (0.06) | 8/1113 (0.7%) | 0.002 (0.007) |
| **mRS at follow up** |  |  |  |  |  |  |
| *6* | 3/220  (1.4%) | 2/89  (2.2%) | 1/131  (0.8%) | 0.35 (0.39) | 81/1284 (6.3%) | 0.003 (0.009) |
| *5* | 14/220  (6.4%) | 3/89  (3.4%) | 11/131  (8.4%) | 0.13 (0.18) | 24/1284  (1.9%) | <0.001 (<0.001) |
| *4* | 47/220  (21.4%) | 10/89  (11.2%) | 37/131  (28.2%) | 0.003 (0.03) | 53/1284  (4.1%) | <0.001 (<0.001) |
| *3* | 55/220  (25.0%) | 22/89  (24.7%) | 33/131  (25.2%) | 0.94 (0.94( | 208/1284  (16.2%) | 0.002 (0.007) |
| *2* | 53/220  (24.1%) | 30/89  (33.7%) | 23/131  (17.6%) | 0.006 (0.03) | 306/1284  (23.8%) | 0.94 (0.94) |
| *1* | 27/220  (12.3%) | 16/89  (18.0%) | 11/131  (8.4%) | 0.03 (0.06) | 238/1284  (18.5%) | 0.02 (0.05) |
| *0* | 21/220  (9.5%) | 6/89  (6.7%) | 15/131  (11.5%) | 0.24 (0.3) | 374/1284  (29.1%) | <0.001 (<0.001) |

**Supplementary table 7** Secondary outcomes

| **Secondary outcomes** | **Total Cohort** | **Adult** | **Paediatric** | ***p value (padj)*** |
| --- | --- | --- | --- | --- |
| Cognitive or memory problems (developmental delay in paediatric cohort) | 153/206  (74.3%) | 63/80  (78.8%) | 90/126  (71.4%) | 0.24 (0.4) |
| Behavioural problems | 42/206  (20.4%) | 18/80  (22.5%) | 24/126  (19.1%) | 0.55 (0.69) |
| Seizures/ Epilepsy | 44/205  (21.5%) | 7/79  (8.86%) | 36/126  (28.6%) | <0.001 (0.002) |
| Motor problems | 65/203  (32.1%) | 9/78  (11.5%) | 56/125  (44.8%) | <0.001 (<0.001) |
| Ongoing ASMs | 45/102  (44.1%) | 19/42  (45.2%) | 26/60  (43.3%) | 0.85 (0.85) |

**Supplementary table 8** Adverse events following immunotherapy

| **Patient ID** | **HSV+ at AE presentation** | **Immunotherapy** | **Adverse Event** | **mRS at follow-up** |
| --- | --- | --- | --- | --- |
| *9* | No | CS, PLEX, IVIG, RTX | Hemodynamic and autonomic instability related to PLEX | 2 |
| *22* | Not reported | CS, PLEX, MMF, RTX | Non-tolerance | 2 |
| *103* | No | IVIG | Development of diabetes insipidus | 1 |
| *181* | Yes | CS PLEX RTX | Septic venous thrombosis | 3 |
| *197* | No | CS | Bowel ischemia due to thrombosis of the mesenteric artery | 6^a^ |
| *208* | No | IVIG RTX | Cholecystitis | 4 |
| *216* | No | CS IVIG | Infections: UTI/LRTI | 4 |

CS = corticosteroids; PLEX = plasma exchange; RTX = rituximab

^a^Cause of death: Bowel Ischemia

**Supplementary table 9** Included studies^a^

| **Reference** | **No. of Patients** | **Year of Publication** |
| --- | --- | --- |
| Ellul MA, Griffiths MJ, Iyer A, et al. Anti-N-methyl-D-aspartate receptor encephalitis in a young child with histological evidence on brain biopsy of coexistent herpes simplex virus type 1 infection. Pediatr Infect Dis J. 2016;35(3):347-349. doi:10.1097/INF.0000000000001011. | 1 | 2016 |
| Leypoldt F, Titulaer MJ, Aguilar E, et al. Herpes simplex virus-1 encephalitis can trigger anti-NMDA receptor encephalitis: case report. Neurology. 2013;81(18):1637-1639. doi:10.1212/WNL.0b013e3182a9f531. | 1 | 2013 |
| Strippel C, Mönig C, Golombeck KS, Dik A, Bönte K, Kovac S, et al. Treating refractory post-herpetic anti-N-methyl-d-aspartate receptor encephalitis with rituximab. Oxf Med Case Reports. 2017 Jul 3;2017(7):omx034. | 1 | 2017 |
| Goenka A, Jain V, Nariai H, Spiro A, Steinschneider M. Extended Clinical Spectrum of Anti-N-Methyl-d-Aspartate Receptor Encephalitis in Children: A Case Series. Pediatr Neurol. 2017;72:51-55. | 1 | 2017 |
| Patil VA, Kulkarni SD, Udwadia-Hegde A, Sayed RJ, Garg M. Anti-N-methyl-D-aspartate receptor encephalitis during relapse of herpes simplex encephalitis in a young boy: A brief review of literature. Neurol India. 2017;65(2):393-397. | 1 | 2017 |
| Pistacchi M, Marsala SZ, Gioulis M, Sanson F, Giometto B. Uncommon relapse after post-herpes simplex encephalitis: an atypical case report. Acta Neurol Belg. 2015;115(4):691-695. | 1 | 2015 |
| Sutcu M, Akturk H, Somer A, et al. Role of Autoantibodies to N-Methyl-d-Aspartate (NMDA) Receptor in Relapsing Herpes Simplex Encephalitis: A Retrospective, One-Center Experience. J Child Neurol. 2016;31(3):345-350. doi:10.1177/0883073815595079 | 1 | 2016 |
| Yushvayev-Cavalier Y, Nichter C, Ramirez-Zamora A. Possible autoimmune association between herpes simplex virus infection and subsequent anti-N-methyl-d-aspartate receptor encephalitis: a pediatric patient with abnormal movements. Pediatr Neurol. 2015;52(4):454-456. doi:10.1016/j.pediatrneurol.2014.10.011 | 1 | 2014 |
| Geoghegan S, Walsh A, King MD, et al. Anti-N-Methyl-D-Aspartate Receptor Antibody Mediated Neurologic Relapse Post Herpes Simplex Encephalitis: A Case Series. Pediatr Infect Dis J. 2016;35(8):e258-e261. doi:10.1097/INF.0000000000001205 | 1 | 2016 |
| Kothur K, Gill D, Wong M, et al. Cerebrospinal fluid cyto-/chemokine profile during acute herpes simplex virus induced anti-N-methyl-d-aspartate receptor encephalitis and in chronic neurological sequelae. Dev Med Child Neurol. 2017;59(8):806-814. doi:10.1111/dmcn.13431 | 1 | 2017 |
| Morris NA, Kaplan TB, Linnoila J, Cho T. HSV encephalitis-induced anti-NMDAR encephalitis in a 67-year-old woman: report of a case and review of the literature. J Neurovirol. 2016;22(1):33-37. doi:10.1007/s13365-015-0364-9 | 1 | 2016 |
| Wickström R, Fowler A, Cooray G, Karlsson-Parra A, Grillner P. Viral triggering of anti-NMDA receptor encephalitis in a child - an important cause for disease relapse. Eur J Paediatr Neurol. 2014;18(4):543-546. doi:10.1016/j.ejpn.2014.03.011 | 1 | 2014 |
| Schein F, Gagneux-Brunon A, Antoine JC, et al. Anti-N-methyl-D-aspartate receptor encephalitis after Herpes simplex virus-associated encephalitis: an emerging disease with diagnosis and therapeutic challenges. Infection. 2017;45(4):545-549. doi:10.1007/s15010-016-0959-y | 1 | 2017 |
| Nosadini M, Mohammad SS, Corazza F, et al. Herpes simplex virus-induced anti-N-methyl-d-aspartate receptor encephalitis: a systematic literature review with analysis of 43 cases. Dev Med Child Neurol. 2017;59(8):796-805. doi:10.1111/dmcn.13448 | 1 | 2017 |
| Mohammad SS, Sinclair K, Pillai S, et al. Herpes simplex encephalitis relapse with chorea is associated with autoantibodies to N-Methyl-D-aspartate receptor or dopamine-2 receptor. Mov Disord. 2014;29(1):117-122. doi:10.1002/mds.25623 | 2 | 2014 |
| Hacohen Y, Deiva K, Pettingill P, et al. N-methyl-D-aspartate receptor antibodies in post-herpes simplex virus encephalitis neurological relapse. Mov Disord. 2014;29(1):90-96. doi:10.1002/mds.25626 | 7 | 2014 |
| Bektaş Ö, Tanyel T, Kocabaş BA, Fitöz S, Ince E, Deda G. Anti-N-methyl-D-aspartate receptor encephalitis that developed after herpes encephalitis: a case report and literature review. Neuropediatrics. 2014;45(6):396-401. doi:10.1055/s-0034-1387813 | 1 | 2014 |
| Desena A, Graves D, Warnack W, Greenberg BM. Herpes simplex encephalitis as a potential cause of anti-N-methyl-D-aspartate receptor antibody encephalitis: report of 2 cases. JAMA Neurol. 2014;71(3):344-346. doi:10.1001/jamaneurol.2013.4580 | 2 | 2014 |
| Bamford A, Crowe BH, Hacohen Y, et al. Pediatric Herpes Simplex Virus Encephalitis Complicated by N-Methyl-D-aspartate Receptor Antibody Encephalitis. J Pediatric Infect Dis Soc. 2015;4(2):e17-e21. doi:10.1093/jpids/piu054 | 1 | 2015 |
| Armangue T, Leypoldt F, Málaga I, et al. Herpes simplex virus encephalitis is a trigger of brain autoimmunity. Ann Neurol. 2014;75(2):317-323. doi:10.1002/ana.24083 | 5 | 2014 |
| Armangue T, Titulaer MJ, Málaga I, et al. Pediatric anti-N-methyl-D-aspartate receptor encephalitis-clinical analysis and novel findings in a series of 20 patients. J Pediatr. 2013;162(4):850-856.e2. doi:10.1016/j.jpeds.2012.10.011 | 4 | 2013 |
| Armangue T, Spatola M, Vlagea A, et al. Frequency, symptoms, risk factors, and outcomes of autoimmune encephalitis after herpes simplex encephalitis: a prospective observational study and retrospective analysis. Lancet Neurol. 2018;17(9):760-772. doi:10.1016/S1474-4422(18)30244-8 | 62 | 2018 |
| Eslamiyeh H, Ranjbar Jamalabadi R, Askarbioki M. Post Herpetic Anti-NMDA- Receptor Encephalitis in an 18-month-old Infant. Iran J Child Neurol. 2023;17(2):167-171. doi:10.22037/ijcn.v17i2.35356 | 1 | 2023 |
| Sahar N, Nurre AM, Simon RQ. Infectious Trigger for Autoimmune Encephalitis: A Case Report and Literature Review. Case Rep Infect Dis. 2019;2019:5731969. Published 2019 Nov 6. doi:10.1155/2019/5731969 | 1 | 2019 |
| Soriano D, Mendoza M, Vélez J, Benavente H, Grosman A. Autoimmune post-herpes simplex encephalitis. A pediatric clinical case report. Encefalitis autoinmune posherpética. Caso clínico pediátrico. Arch Argent Pediatr. 2023;121(6):e202202941. doi:10.5546/aap.2022-02941.eng | 1 | 2023 |
| Kaneko A, Kaneko J, Tominaga N, et al. Pitfalls in clinical diagnosis of anti-NMDA receptor encephalitis. J Neurol. 2018;265(3):586-596. doi:10.1007/s00415-018-8749-3 | 1 | 2018 |
| Toro J, Rivera JS, Moutran-Barroso H, Valencia-Enciso N. Acute HSV and anti-NMDA encephalitis occurring as a neurosurgical complication. BMJ Case Rep. 2021;14(5):e241136. Published 2021 May 26. doi:10.1136/bcr-2020-241136 | 1 | 2021 |
| Erkent I, Gocmen R, Tezer FI, et al. Postherpetic Anti-N-methyl-D-aspartate Receptor Encephalitis after Hemispherotomy in a Patient with Intractable Startle Epilepsy. Neuropediatrics. 2018;49(1):63-67. doi:10.1055/s-0037-1606640 | 1 | 2018 |
| Al Futaisi A, Uraba WB, Al Dhawi N, et al. N-Methyl-D-Aspartate Receptor Encephalitis, Post Herpes Encephalitis in Two Pediatric Cases. Oman Med J. 2023;38(6):e578. Published 2023 Nov 30. doi:10.5001/omj.2023.68 | 1 | 2023 |
| Sun S, Ren J, Zhong Z, et al. Case report: Overlapping anti-AMPAR encephalitis with anti-IgLON5 disease post herpes simplex virus encephalitis. Front Immunol. 2024;14:1329540. Published 2024 Jan 8. doi:10.3389/fimmu.2023.1329540 | 1 | 2024 |
| Zhang J, Liu J, Wang J, Gan J. Anti-NMDAR encephalitis secondary to acute necrotizing encephalopathy caused by herpes simplex virus infection in infants: Case series. Clin Neurol Neurosurg. 2023;233:107955. doi:10.1016/j.clineuro.2023.107955 | 4 | 2023 |
| Lee J, Kelly R, Kobayashi ES. Acute neurologic changes in a 13-month-old with a history of neonatal HSV and variant in RNF213. Crit Care Med. 2024;52(1 Suppl 1):S384. | 1 | 2024 |
| Haddad A, Rambaud T, Tran Ba S, Manceau P, Degos B, Mongin M. Anti-NMDAR encephalitis following herpes simplex encephalitis: A case report and update on diagnostic and treatment. Rev Neurol. 2022;178(10):1107-1109. | 1 | 2022 |
| Tailland M, Le Verger L, Honnorat J, Biquard F, Codron P, Cassereau J. Post-herpetic anti-N-methyl-d-aspartate receptor encephalitis in a pregnant woman. Rev Neurol (Paris). 2020;176(1-2):129-131. doi:10.1016/j.neurol.2019.01.402 | 1 | 2020 |
| Peters J, Wesley SF. Case of concurrent herpes simplex and autoimmune encephalitis. Neurol Neuroimmunol Neuroinflamm. 2020;7(6):e897. Published 2020 Oct 2. doi:10.1212/NXI.0000000000000897 | 1 | 2020 |
| Quade A, Rostasy K, Wickström R, et al. Autoimmune Encephalitis with Autoantibodies to NMDAR1 following Herpes Encephalitis in Children and Adolescents. Neuropediatrics. 2023;54(1):14-19. doi:10.1055/s-0042-1757706 | 15 | 2023 |
| Whinnery C, Cinderella MA, Nash R. When zebras travel in packs: HSV and anti-NMDA receptor encephalitis with secondary delirious catatonic mania. J Acad Consult Liaison Psychiatry. 2022;63(Suppl 2):S163-S164. | 1 | 2022 |
| Ho AC, Chan SH, Chan E, et al. Anti-N-methyl-d-aspartate receptor encephalitis in children: Incidence and experience in Hong Kong. Brain Dev. 2018;40(6):473-479. | 1 | 2018 |
| Ayvacioglu Cagan C, Gocmen R, Acar Ozen NP, Tuncer A. Life after tetra hit: Anti-NMDAR encephalitis after HSV encephalitis in a NMOSD coexistent with Sjogren's syndrome. Noro Psikiyatr Ars. 2022;59(2):161-163. | 1 | 2022 |
| Yan Hung SK, Hiew FL, Viswanathan S. Anti-NMDAR encephalitis in association with herpes simplex virus and viral and bacterial zoonoses. Ann Indian Acad Neurol. 2019;22(1):102-103. | 1 | 2019 |
| Ko JM, Kim WJ, Kim SY, et al. Hyperammonemia in a case of herpes simplex and anti-N-methyl-d-aspartate receptor encephalitis. Brain Dev. 2019;41(7):634-637. | 1 | 2019 |
| Klein da Costa B, de Oliveira Pinto P, Staub L, et al. Neurological syndromes and potential triggers associated with antibodies to neuronal surface antigens. Mult Scler Relat Disord. 2023;80:105022. | 3 | 2023 |
| Gomez DF, Gomez JM, Jaramillo-Velasquez D, Hakim F, Pedraza C, Reyes E. The first recorded case of herpes simplex virus encephalitis followed by anti-NMDA receptor autoimmune encephalitis after resection of meningioma. Interdiscip Neurosurg. 2021;24:101046. | 1 | 2021 |
| Kornbluh AB, McLendon L, Banwell B. Anti-N-methyl-D-aspartic acid receptor encephalitis after recurrent herpes simplex infection: A case report and literature review. Neuroimmunol Rep. 2022;2:100147. | 1 | 2022 |
| Chen S, Ren H, Lin F, Fan S, Cao Y, Zhao W, Guan H. Anti-metabotropic glutamate receptor 5 encephalitis: Five case reports and literature review. Brain Behav. 2023;13(5):e3003. | 1 | 2023 |
| Carrascosa-Garcia P, Oviedo-Melgares L, Torres-Fernandez D, et al. Rituximab as second-line treatment in anti-NMDAR encephalitis after herpes simplex encephalitis in children. Indian J Pediatr. 2022;89(10):1031-1033. | 2 | 2022 |
| Narasimhappa K, Mamtani H, Jain K, et al. Unmasking bipolarity in recurrent depressive disorder following herpes simplex virus triggered n-methyl-D-aspartate encephalitis. Bipolar Disord. 2024;26(2):192-195. | 1 | 2024 |
| Dorcet G, Benaiteau M, Bost C, et al. Two cases of late-onset anti-NMDAr auto-immune encephalitis after herpes simplex virus 1 encephalitis. Front Neurol. 2020;11:38. | 2 | 2020 |
| Roome K, Tutmaher M, Jenkins E, Orenstein E, Gombolay G. A case of lethargy followed by new abnormal movements in an infant with a history of HSV encephalitis. Clin Pediatr (Phila). 2022;61(9):645-648. | 1 | 2022 |
| Jiang Y, Yao Z, Jiang L, Tan C. Virus reactivation after immunotherapy of anti-NMDAR encephalitis secondary to herpes simplex encephalitis: A case report. Brain Dev. 2021;43(10):1057-1060. | 1 | 2021 |
| Armangue T, Baucells BJ, Vlagea A, et al. Toll-like receptor 3 deficiency in autoimmune encephalitis post-herpes simplex encephalitis. Neurol Neuroimmunol Neuroinflamm. 2019;6(6):e611. | 1 | 2019 |
| Alexopoulos H, Akrivou S, Mastroyanni S, et al. Postherpes simplex encephalitis: a case series of viral-triggered autoimmunity, synaptic autoantibodies and response to therapy. Ther Adv Neurol Disord. 2018;11:1756286418768778. | 5 | 2018 |
| Manglani M, Poley M, Kumar A, McSherry G, Ericson JE. Anti-NMDAR encephalitis after neonatal HSV-1 infection in a child with low TLR-3 function. Pediatrics. 2021;148(3):e2020035824. | 1 | 2021 |
| Tay MRJ, Yeo T, Chen Z, Au WL, Tan K. Movement disorders in an adult patient with anti-NMDAR encephalitis after herpes simplex encephalitis. Mov Disord Clin Pract. 2017;4(3):460-462. | 1 | 2017 |
| Handoko M, Hong W, Espineli E, Saxena K, Muscal E, Risen S. Autoimmune glial fibrillary acidic protein astrocytopathy following herpes simplex virus encephalitis in a pediatric patient. Pediatr Neurol. 2019;98:85-86. | 1 | 2019 |
| Brás A, André A, Sá L, et al. Anti-NMDAR encephalitis following herpes simplex virus encephalitis: 2 cases from Portugal. Neurohospitalist. 2020;10(2):133-138. | 2 | 2020 |
| Omae T, Saito Y, Tsuchie H, Ohno K, Maegaki Y, Sakuma H. Cytokine/chemokine elevation during the transition phase from HSV encephalitis to autoimmune anti-NMDA receptor encephalitis. Brain Dev. 2018;40(4):361-365. | 1 | 2018 |
| Hu S, Lan T, Bai R, Jiang S, Cai J, Ren L. HSV encephalitis triggered anti-NMDAR encephalitis: a case report. Neurol Sci. 2021;42(3):857-861. | 1 | 2021 |
| Berek K, Beer R, Grams A, et al. Caspr2 antibodies in herpes simplex encephalitis: an extension of the spectrum of virus induced autoimmunity? - A case report. BMC Neurol. 2022;22(1):131. | 1 | 2022 |
| Marcus L, Ness JM. Pediatric N-methyl-d-aspartate (NMDA) receptor encephalitis, with and without herpes encephalitis. J Child Neurol. 2021;36(9):743-751. | 6 | 2021 |
| Ramesh V, Sankar J. Autoimmune encephalitis following herpes simplex virus encephalitis in an infant. Indian Pediatr. 2018;55(3):260. | 1 | 2018 |
| Epstein S, Ankam J, Vargas WS, Thakur KT. Critical analysis of a challenging case of post-infectious N-methyl-D-aspartate receptor encephalitis. Neurohospitalist. 2021;11(2):160-164. | 1 | 2021 |
| Armangue T, Moris G, Cantarín-Extremera V, et al. Autoimmune post-herpes simplex encephalitis of adults and teenagers. Neurology. 2015;85(20):1736-1743. | 8 | 2015 |
| Li J, Xu Y, Ren H, Zhu Y, Peng B, Cui L. Autoimmune GFAP astrocytopathy after viral encephalitis: A case report. Mult Scler Relat Disord. 2018;21:84-87. | 1 | 2018 |
| Mrad L, Moustakas A, Fuino R, Waheed W. Severe presentation of antibody-negative, postinfectious steroid-responsive encephalitis and atonic bladder after herpes simplex encephalitis. BMJ Case Rep. 2019;12(7):e230005. doi:10.1136/bcr-2019-230005. | 1 | 2019 |
| Sutcu M, Akturk H, Somer A, et al. Role of autoantibodies to N-methyl-d-aspartate (NMDA) receptor in relapsing herpes simplex encephalitis: a retrospective, one-center experience. J Child Neurol. 2016;31(3):345-350. | 1 | 2016 |
| Geoghegan S, Walsh A, King MD, et al. Anti-N-methyl-D-aspartate receptor antibody mediated neurologic relapse post herpes simplex encephalitis: a case series. Pediatr Infect Dis J. 2016;35(8):e258-261. | 2 | 2016 |
| Sandweiss AJ, Erickson TA, Jiang Y, et al. Infectious profiles in pediatric anti-N-methyl-D-aspartate receptor encephalitis. J Neuroimmunol. 2023;381:578139. | 11 | 2023 |
| Gadoth A, Segal Y, Paran Y, Aizenstein O, Alcalay Y. The importance of tissue-based assay in the diagnosis of autoimmune encephalitis. J Neurol. 2022;269(7):3588-3596. doi:10.1007/s00415-022-10973-8. | 1 | 2022 |
| Vasireddy RP, Guduru ZG, Khan QK. A case series of children with choreo-athetoid movements diagnosed with anti-N-methyl-D-aspartate receptor encephalitis [abstract]. Mov Disord. 2022;37(suppl 2). | 1 | 2024 |
| Wanigasinghe J, Anuradha KWDA, Chang T. Pediatric autoimmune encephalitis in Sri Lanka: a single-center experience over 7 years. J Pediatr Neurol. 2022;20(6):373-379. doi:10.1055/s-0041-1739260 | 1 | 2022 |
| Schuster S, Abrante L, Matschke J, et al. Fatal PCR-negative herpes simplex virus-1 encephalitis with GABA(A) receptor antibodies. Neurol Neuroimmunol Neuroinflamm. 2019;6(6):e624. doi:10.1212/NXI.0000000000000624 | 1 | 2019 |
| Kolla S, Balleda L, Thimmapuram CR. Anti-N-methyl-D-Aspartate-Receptor encephalitis following herpes simplex virus encephalitis - Presenting as a pediatric patient with abnormal movements and psychiatric manifestation. Indian J Med Spec. 2023;14(1):56-59. doi:10.4103/injms.injms_127_22 | 1 | 2023 |
| Dumez P, Villagrán-García M, Bani-Sadr A, et al. Specific clinical and radiological characteristics of anti-NMDA receptor autoimmune encephalitis following herpes encephalitis. J Neurol. Published online August 16, 2024. doi:10.1007/s00415-024-12615-7. | 13 | 2024 |
| Swayne A, Warren N, Prain K, Gillis D, Wong R, Blum S. Analysing triggers for anti-NMDA-receptor encephalitis including herpes simplex virus encephalitis and ovarian teratoma: results from the Queensland Autoimmune Encephalitis cohort. Intern Med J. 2022;52(11):1943-1949. doi:10.1111/imj.15472. | 3 | 2022 |
| Søgaard A, Poulsen CA, Belhouche NZ, et al. Post-herpetic anti-NMDAR encephalitis in Denmark: current status and future challenges. Biomedicines. 2024;12(9):1953. doi:10.3390/biomedicines12091953. | 8 | 2024 |

^a^Additional data extracted from a cohort of 3 patients with HSVE-AE from the affiliation of the corresponding author

**Supplementary table 10** JBI critical appraisal tool: case reports

| **Reference** | **No. of Patients** | **Year of Publication** | **Were patient’s demographic characteristics clearly described?** | **Was the patient’s history clearly described and presented as a timeline?** | **Was the current clinical condition of the patient on presentation clearly described?** | **Were diagnostic tests or assessment methods and the results clearly described?** | **Was the intervention(s) or treatment procedure(s) clearly described?** | **Was the post-intervention clinical condition clearly described?** | **Were adverse events (harms) or unanticipated events identified and described?** | **Does the case report provide takeaway lessons?** | **Include or Exclude (1/0)** | **Received Additional Data from Authors** |
| --- | --- | --- | --- | --- | --- | --- | --- | --- | --- | --- | --- | --- |
| Ellul MA, Griffiths MJ, Iyer A, et al. Anti-N-methyl-D-aspartate receptor encephalitis in a young child with histological evidence on brain biopsy of coexistent herpes simplex virus type 1 infection. Pediatr Infect Dis J. 2016;35(3):347-349. doi:10.1097/INF.0000000000001011. | 1 | 2016 | 1 | 1 | 1 | 1 | 1 | 1 | 0 | 1 | 1 | NA |
| Leypoldt F, Titulaer MJ, Aguilar E, et al. Herpes simplex virus-1 encephalitis can trigger anti-NMDA receptor encephalitis: case report. Neurology. 2013;81(18):1637-1639. doi:10.1212/WNL.0b013e3182a9f531. | 1 | 2013 | 1 | 0 | 1 | 1 | 1 | 1 | 0 | 1 | 1 | NA |
| Strippel C, Mönig C, Golombeck KS, Dik A, Bönte K, Kovac S, et al. Treating refractory post-herpetic anti-N-methyl-d-aspartate receptor encephalitis with rituximab. Oxf Med Case Reports. 2017 Jul 3;2017(7):omx034. | 1 | 2017 | 1 | 1 | 1 | 1 | 1 | 1 | 0 | 1 | 1 | NA |
| Patil VA, Kulkarni SD, Udwadia-Hegde A, Sayed RJ, Garg M. Anti-N-methyl-D-aspartate receptor encephalitis during relapse of herpes simplex encephalitis in a young boy: A brief review of literature. Neurol India. 2017;65(2):393-397. | 1 | 2017 | 1 | 1 | 1 | 1 | 1 | 1 | 0 | 1 | 1 | NA |
| Pistacchi M, Marsala SZ, Gioulis M, Sanson F, Giometto B. Uncommon relapse after post-herpes simplex encephalitis: an atypical case report. Acta Neurol Belg. 2015;115(4):691-695. | 1 | 2015 | 1 | 0 | 1 | 1 | 1 | 1 | 0 | 1 | 1 | NA |
| Yushvayev-Cavalier Y, Nichter C, Ramirez-Zamora A. Possible autoimmune association between herpes simplex virus infection and subsequent anti-N-methyl-d-aspartate receptor encephalitis: a pediatric patient with abnormal movements. Pediatr Neurol. 2015;52(4):454-456. doi:10.1016/j.pediatrneurol.2014.10.011 | 1 | 2014 | 1 | 0 | 1 | 1 | 1 | 1 | 0 | 1 | 1 | NA |
| Kothur K, Gill D, Wong M, et al. Cerebrospinal fluid cyto-/chemokine profile during acute herpes simplex virus induced anti-N-methyl-d-aspartate receptor encephalitis and in chronic neurological sequelae. Dev Med Child Neurol. 2017;59(8):806-814. doi:10.1111/dmcn.13431 | 1 | 2017 | 1 | 0 | 1 | 1 | 1 | 1 | 0 | 1 | 1 | NA |
| Morris NA, Kaplan TB, Linnoila J, Cho T. HSV encephalitis-induced anti-NMDAR encephalitis in a 67-year-old woman: report of a case and review of the literature. J Neurovirol. 2016;22(1):33-37. doi:10.1007/s13365-015-0364-9 | 1 | 2016 | 1 | 0 | 1 | 1 | 1 | 1 | 0 | 1 | 1 | NA |
| Wickström R, Fowler A, Cooray G, Karlsson-Parra A, Grillner P. Viral triggering of anti-NMDA receptor encephalitis in a child - an important cause for disease relapse. Eur J Paediatr Neurol. 2014;18(4):543-546. doi:10.1016/j.ejpn.2014.03.011 | 1 | 2014 | 1 | 1 | 1 | 1 | 1 | 1 | 0 | 0 | 1 | NA |
| Schein F, Gagneux-Brunon A, Antoine JC, et al. Anti-N-methyl-D-aspartate receptor encephalitis after Herpes simplex virus-associated encephalitis: an emerging disease with diagnosis and therapeutic challenges. Infection. 2017;45(4):545-549. doi:10.1007/s15010-016-0959-y | 1 | 2017 | 1 | 0 | 1 | 1 | 1 | 1 | 0 | 1 | 1 | NA |
| Nosadini M, Mohammad SS, Corazza F, et al. Herpes simplex virus-induced anti-N-methyl-d-aspartate receptor encephalitis: a systematic literature review with analysis of 43 cases. Dev Med Child Neurol. 2017;59(8):796-805. doi:10.1111/dmcn.13448 | 1 | 2017 | 1 | 1 | 1 | 1 | 1 | 1 | 0 | 1 | 1 | NA |
| Bektaş Ö, Tanyel T, Kocabaş BA, Fitöz S, Ince E, Deda G. Anti-N-methyl-D-aspartate receptor encephalitis that developed after herpes encephalitis: a case report and literature review. Neuropediatrics. 2014;45(6):396-401. doi:10.1055/s-0034-1387813 | 1 | 2014 | 1 | 0 | 1 | 1 | 1 | 1 | 0 | 1 | 1 | NA |
| Bamford A, Crowe BH, Hacohen Y, et al. Pediatric Herpes Simplex Virus Encephalitis Complicated by N-Methyl-D-aspartate Receptor Antibody Encephalitis. J Pediatric Infect Dis Soc. 2015;4(2):e17-e21. doi:10.1093/jpids/piu054 | 1 | 2015 | 1 | 0 | 1 | 1 | 1 | 1 | 0 | 1 | 1 | NA |
| Eslamiyeh H, Ranjbar Jamalabadi R, Askarbioki M. Post Herpetic Anti-NMDA- Receptor Encephalitis in an 18-month-old Infant. Iran J Child Neurol. 2023;17(2):167-171. doi:10.22037/ijcn.v17i2.35356 | 1 | 2023 | 1 | 0 | 1 | 1 | 1 | 1 | 0 | 1 | 1 | NA |
| Sahar N, Nurre AM, Simon RQ. Infectious Trigger for Autoimmune Encephalitis: A Case Report and Literature Review. Case Rep Infect Dis. 2019;2019:5731969. Published 2019 Nov 6. doi:10.1155/2019/5731969 | 1 | 2019 | 1 | 1 | 1 | 1 | 1 | 1 | 0 | 1 | 1 | NA |
| Soriano D, Mendoza M, Vélez J, Benavente H, Grosman A. Autoimmune post-herpes simplex encephalitis. A pediatric clinical case report. Encefalitis autoinmune posherpética. Caso clínico pediátrico. Arch Argent Pediatr. 2023;121(6):e202202941. doi:10.5546/aap.2022-02941.eng | 1 | 2023 | 1 | 1 | 1 | 1 | 1 | 1 | 0 | 1 | 1 | NA |
| Toro J, Rivera JS, Moutran-Barroso H, Valencia-Enciso N. Acute HSV and anti-NMDA encephalitis occurring as a neurosurgical complication. BMJ Case Rep. 2021;14(5):e241136. Published 2021 May 26. doi:10.1136/bcr-2020-241136 | 1 | 2021 | 1 | 0 | 1 | 1 | 1 | 1 | 1 | 1 | 1 | NA |
| Erkent I, Gocmen R, Tezer FI, et al. Postherpetic Anti-N-methyl-D-aspartate Receptor Encephalitis after Hemispherotomy in a Patient with Intractable Startle Epilepsy. Neuropediatrics. 2018;49(1):63-67. doi:10.1055/s-0037-1606640 | 1 | 2018 | 1 | 0 | 1 | 1 | 1 | 1 | 0 | 1 | 1 | NA |
| Al Futaisi A, Uraba WB, Al Dhawi N, et al. N-Methyl-D-Aspartate Receptor Encephalitis, Post Herpes Encephalitis in Two Pediatric Cases. Oman Med J. 2023;38(6):e578. Published 2023 Nov 30. doi:10.5001/omj.2023.68 | 1 | 2023 | 1 | 0 | 1 | 1 | 1 | 1 | 0 | 1 | 1 | NA |
| Sun S, Ren J, Zhong Z, et al. Case report: Overlapping anti-AMPAR encephalitis with anti-IgLON5 disease post herpes simplex virus encephalitis. Front Immunol. 2024;14:1329540. Published 2024 Jan 8. doi:10.3389/fimmu.2023.1329540 | 1 | 2024 | 1 | 1 | 1 | 1 | 1 | 1 | 0 | 1 | 1 | NA |
| Lee J, Kelly R, Kobayashi ES. Acute neurologic changes in a 13-month-old with a history of neonatal HSV and variant in RNF213. Crit Care Med. 2024;52(1 Suppl 1):S384. | 1 | 2024 | 1 | 0 | 1 | 0 | 1 | 1 | 0 | 1 | 1 | NA |
| Haddad A, Rambaud T, Tran Ba S, Manceau P, Degos B, Mongin M. Anti-NMDAR encephalitis following herpes simplex encephalitis: A case report and update on diagnostic and treatment. Rev Neurol. 2022;178(10):1107-1109. | 1 | 2022 | 1 | 0 | 1 | 1 | 1 | 1 | 0 | 1 | 1 | NA |
| Tailland M, Le Verger L, Honnorat J, Biquard F, Codron P, Cassereau J. Post-herpetic anti-N-methyl-d-aspartate receptor encephalitis in a pregnant woman. Rev Neurol (Paris). 2020;176(1-2):129-131. doi:10.1016/j.neurol.2019.01.402 | 1 | 2020 | 1 | 0 | 1 | 1 | 1 | 1 | 0 | 1 | 1 | NA |
| Peters J, Wesley SF. Case of concurrent herpes simplex and autoimmune encephalitis. Neurol Neuroimmunol Neuroinflamm. 2020;7(6):e897. Published 2020 Oct 2. doi:10.1212/NXI.0000000000000897 | 1 | 2020 | 1 | 1 | 1 | 1 | 1 | 1 | 0 | 1 | 1 | NA |
| Whinnery C, Cinderella MA, Nash R. When zebras travel in packs: HSV and anti-NMDA receptor encephalitis with secondary delirious catatonic mania. J Acad Consult Liaison Psychiatry. 2022;63(Suppl 2):S163-S164. | 1 | 2022 | 1 | 0 | 1 | 1 | 1 | 1 | 0 | 1 | 1 | NA |
| Ho AC, Chan SH, Chan E, et al. Anti-N-methyl-d-aspartate receptor encephalitis in children: Incidence and experience in Hong Kong. Brain Dev. 2018;40(6):473-479. | 1 | 2018 | 1 | 0 | 1 | 1 | 1 | 1 | 0 | 1 | 1 | NA |
| Ayvacioglu Cagan C, Gocmen R, Acar Ozen NP, Tuncer A. Life after tetra hit: Anti-NMDAR encephalitis after HSV encephalitis in a NMOSD coexistent with Sjogren's syndrome. Noro Psikiyatr Ars. 2022;59(2):161-163. | 1 | 2022 | 1 | 0 | 1 | 1 | 1 | 1 | 0 | 1 | 1 | NA |
| Yan Hung SK, Hiew FL, Viswanathan S. Anti-NMDAR encephalitis in association with herpes simplex virus and viral and bacterial zoonoses. Ann Indian Acad Neurol. 2019;22(1):102-103. | 1 | 2019 | 1 | 0 | 1 | 1 | 1 | 1 | 0 | 1 | 1 | NA |
| Ko JM, Kim WJ, Kim SY, et al. Hyperammonemia in a case of herpes simplex and anti-N-methyl-d-aspartate receptor encephalitis. Brain Dev. 2019;41(7):634-637. | 1 | 2019 | 1 | 0 | 1 | 1 | 1 | 1 | 0 | 1 | 1 | NA |
| Gomez DF, Gomez JM, Jaramillo-Velasquez D, Hakim F, Pedraza C, Reyes E. The first recorded case of herpes simplex virus encephalitis followed by anti-NMDA receptor autoimmune encephalitis after resection of meningioma. Interdiscip Neurosurg. 2021;24:101046. | 1 | 2021 | 1 | 0 | 1 | 1 | 1 | 1 | 0 | 1 | 1 | NA |
| Kornbluh AB, McLendon L, Banwell B. Anti-N-methyl-D-aspartic acid receptor encephalitis after recurrent herpes simplex infection: A case report and literature review. Neuroimmunol Rep. 2022;2:100147. | 1 | 2022 | 1 | 0 | 1 | 1 | 1 | 1 | 0 | 0 | 1 | NA |
| Narasimhappa K, Mamtani H, Jain K, et al. Unmasking bipolarity in recurrent depressive disorder following herpes simplex virus triggered n-methyl-D-aspartate encephalitis. Bipolar Disord. 2024;26(2):192-195. | 1 | 2024 | 1 | 0 | 1 | 1 | 1 | 1 | 0 | 1 | 1 | NA |
| Roome K, Tutmaher M, Jenkins E, Orenstein E, Gombolay G. A case of lethargy followed by new abnormal movements in an infant with a history of HSV encephalitis. Clin Pediatr (Phila). 2022;61(9):645-648. | 1 | 2022 | 1 | 0 | 1 | 1 | 1 | 1 | 0 | 1 | 1 | NA |
| Jiang Y, Yao Z, Jiang L, Tan C. Virus reactivation after immunotherapy of anti-NMDAR encephalitis secondary to herpes simplex encephalitis: A case report. Brain Dev. 2021;43(10):1057-1060. | 1 | 2021 | 1 | 0 | 1 | 1 | 1 | 1 | 0 | 1 | 1 | NA |
| Armangue T, Baucells BJ, Vlagea A, et al. Toll-like receptor 3 deficiency in autoimmune encephalitis post-herpes simplex encephalitis. Neurol Neuroimmunol Neuroinflamm. 2019;6(6):e611. | 1 | 2019 | 1 | 1 | 1 | 1 | 1 | 1 | 0 | 1 | 1 | 0 |
| Manglani M, Poley M, Kumar A, McSherry G, Ericson JE. Anti-NMDAR encephalitis after neonatal HSV-1 infection in a child with low TLR-3 function. Pediatrics. 2021;148(3):e2020035824. | 1 | 2021 | 1 | 0 | 1 | 1 | 1 | 1 | 0 | 1 | 1 | NA |
| Tay MRJ, Yeo T, Chen Z, Au WL, Tan K. Movement disorders in an adult patient with anti-NMDAR encephalitis after herpes simplex encephalitis. Mov Disord Clin Pract. 2017;4(3):460-462. | 1 | 2017 | 1 | 0 | 1 | 1 | 1 | 1 | 0 | 1 | 1 | NA |
| Handoko M, Hong W, Espineli E, Saxena K, Muscal E, Risen S. Autoimmune glial fibrillary acidic protein astrocytopathy following herpes simplex virus encephalitis in a pediatric patient. Pediatr Neurol. 2019;98:85-86. | 1 | 2019 | 1 | 0 | 1 | 1 | 1 | 1 | 0 | 1 | 1 | NA |
| Omae T, Saito Y, Tsuchie H, Ohno K, Maegaki Y, Sakuma H. Cytokine/chemokine elevation during the transition phase from HSV encephalitis to autoimmune anti-NMDA receptor encephalitis. Brain Dev. 2018;40(4):361-365. | 1 | 2018 | 1 | 1 | 1 | 1 | 1 | 1 | 0 | 1 | 1 | NA |
| Hu S, Lan T, Bai R, Jiang S, Cai J, Ren L. HSV encephalitis triggered anti-NMDAR encephalitis: a case report. Neurol Sci. 2021;42(3):857-861. | 1 | 2021 | 1 | 1 | 1 | 1 | 1 | 1 | 0 | 1 | 1 | NA |
| Berek K, Beer R, Grams A, et al. Caspr2 antibodies in herpes simplex encephalitis: an extension of the spectrum of virus induced autoimmunity? - A case report. BMC Neurol. 2022;22(1):131. | 1 | 2022 | 1 | 1 | 1 | 1 | 1 | 1 | 0 | 1 | 1 | NA |
| Ramesh V, Sankar J. Autoimmune encephalitis following herpes simplex virus encephalitis in an infant. Indian Pediatr. 2018;55(3):260. | 1 | 2018 | 1 | 1 | 1 | 1 | 1 | 1 | 0 | 1 | 1 | NA |
| Epstein S, Ankam J, Vargas WS, Thakur KT. Critical analysis of a challenging case of post-infectious N-methyl-D-aspartate receptor encephalitis. Neurohospitalist. 2021;11(2):160-164. | 1 | 2021 | 1 | 1 | 1 | 1 | 1 | 1 | 0 | 1 | 1 | NA |
| Li J, Xu Y, Ren H, Zhu Y, Peng B, Cui L. Autoimmune GFAP astrocytopathy after viral encephalitis: A case report. Mult Scler Relat Disord. 2018;21:84-87. | 1 | 2018 | 1 | 1 | 1 | 1 | 1 | 1 | 0 | 1 | 1 | 0 |
| Mrad L, Moustakas A, Fuino R, Waheed W. Severe presentation of antibody-negative, postinfectious steroid-responsive encephalitis and atonic bladder after herpes simplex encephalitis. BMJ Case Rep. 2019;12(7):e230005. doi:10.1136/bcr-2019-230005. | 1 | 2019 | 1 | 1 | 1 | 1 | 1 | 1 | 0 | 1 | 1 | NA |
| Gadoth A, Segal Y, Paran Y, Aizenstein O, Alcalay Y. The importance of tissue-based assay in the diagnosis of autoimmune encephalitis. J Neurol. 2022;269(7):3588-3596. doi:10.1007/s00415-022-10973-8. | 1 | 2022 | 1 | 1 | 1 | 1 | 1 | 1 | 1 | 1 | 1 | 1 |
| Vasireddy RP, Guduru ZG, Khan QK. A case series of children with choreo-athetoid movements diagnosed with anti-N-methyl-D-aspartate receptor encephalitis [abstract]. Mov Disord. 2022;37(suppl 2). | 1 | 2024 | 1 | 0 | 1 | 1 | 1 | 1 | 0 | 1 | 1 | NA |
| Wanigasinghe J, Anuradha KWDA, Chang T. Pediatric autoimmune encephalitis in Sri Lanka: a single-center experience over 7 years. J Pediatr Neurol. 2022;20(6):373-379. doi:10.1055/s-0041-1739260 | 1 | 2022 | 1 | 1 | 1 | 1 | 1 | 1 | 1 | 1 | 1 | 1 |
| Schuster S, Abrante L, Matschke J, et al. Fatal PCR-negative herpes simplex virus-1 encephalitis with GABA(A) receptor antibodies. Neurol Neuroimmunol Neuroinflamm. 2019;6(6):e624. doi:10.1212/NXI.0000000000000624 | 1 | 2019 | 1 | 1 | 1 | 1 | 1 | 1 | 1 | 1 | 1 | NA |
| Kolla S, Balleda L, Thimmapuram CR. Anti-N-methyl-D-Aspartate-Receptor encephalitis following herpes simplex virus encephalitis - Presenting as a pediatric patient with abnormal movements and psychiatric manifestation. Indian J Med Spec. 2023;14(1):56-59. doi:10.4103/injms.injms_127_22 | 1 | 2023 | 1 | 1 | 1 | 1 | 1 | 1 | 0 | 1 | 1 | NA |
| Goenka A, Jain V, Nariai H, Spiro A, Steinschneider M. Extended Clinical Spectrum of Anti-N-Methyl-d-Aspartate Receptor Encephalitis in Children: A Case Series. Pediatr Neurol. 2017;72:51-55. | 1 | 2017 | 1 | 1 | 1 | 1 | 1 | 1 | 0 | 1 | 1 | NA |
| Sutcu M, Akturk H, Somer A, et al. Role of Autoantibodies to N-Methyl-d-Aspartate (NMDA) Receptor in Relapsing Herpes Simplex Encephalitis: A Retrospective, One-Center Experience. J Child Neurol. 2016;31(3):345-350. doi:10.1177/0883073815595079 | 1 | 2016 | 1 | 1 | 1 | 1 | 1 | 1 | 0 | 1 | 1 | NA |
| Chen S, Ren H, Lin F, Fan S, Cao Y, Zhao W, Guan H. Anti-metabotropic glutamate receptor 5 encephalitis: Five case reports and literature review. Brain Behav. 2023;13(5):e3003. | 1 | 2023 | 1 | 1 | 1 | 1 | 1 | 1 | 0 | 0 | 1 | NA |

**Supplementary table 11** JBI critical appraisal tool: case series

| **Reference** | **No. of Patients** | **Year of Publication** | **Clear inclusion criteria?** | **Standard measurements?** | **Valid methods for all?** | **Consecutive inclusion of participants?** | **Complete inclusion of participants?** | **Demographics reported?** | **Clear clinical information reporting?** | **Outcomes clearly reported?** | **Presenting site(s) demographic information reported?** | **Appropriate statistical analysis?** | **Include?** | **Received additional data from authors** |
| --- | --- | --- | --- | --- | --- | --- | --- | --- | --- | --- | --- | --- | --- | --- |
| Geoghegan S, Walsh A, King MD, et al. Anti-N-Methyl-D-Aspartate Receptor Antibody Mediated Neurologic Relapse Post Herpes Simplex Encephalitis: A Case Series. Pediatr Infect Dis J. 2016;35(8):e258-e261. doi:10.1097/INF.0000000000001205 | 3 | 2016 | 1 | 1 | 1 | Unclear | 1 | 1 | 1 | 1 | 0 | NA | 1 | NA |
| Mohammad SS, Sinclair K, Pillai S, et al. Herpes simplex encephalitis relapse with chorea is associated with autoantibodies to N-Methyl-D-aspartate receptor or dopamine-2 receptor. Mov Disord. 2014;29(1):117-122. doi:10.1002/mds.25623 | 2 | 2014 | 1 | 1 | 1 | Unclear | 1 | 1 | 1 | 1 | 0 | NA | 1 | NA |
| Hacohen Y, Deiva K, Pettingill P, et al. N-methyl-D-aspartate receptor antibodies in post-herpes simplex virus encephalitis neurological relapse. Mov Disord. 2014;29(1):90-96. doi:10.1002/mds.25626 | 7 | 2014 | 1 | 1 | 1 | Unclear | 1 | 1 | 1 | 1 | 0 | NA | 1 | NA |
| Desena A, Graves D, Warnack W, Greenberg BM. Herpes simplex encephalitis as a potential cause of anti-N-methyl-D-aspartate receptor antibody encephalitis: report of 2 cases. JAMA Neurol. 2014;71(3):344-346. doi:10.1001/jamaneurol.2013.4580 | 2 | 2014 | 1 | 1 | 1 | Unclear | 1 | 1 | 1 | 1 | 0 | NA | 1 | NA |
| Armangue T, Leypoldt F, Málaga I, et al. Herpes simplex virus encephalitis is a trigger of brain autoimmunity. Ann Neurol. 2014;75(2):317-323. doi:10.1002/ana.24083 | 5 | 2014 | 1 | 1 | 1 | Unclear | 1 | 1 | 1 | 1 | 1 | 1 | 1 | NA |
| Armangue T, Titulaer MJ, Málaga I, et al. Pediatric anti-N-methyl-D-aspartate receptor encephalitis-clinical analysis and novel findings in a series of 20 patients. J Pediatr. 2013;162(4):850-856.e2. doi:10.1016/j.jpeds.2012.10.011 | 4 | 2013 | 1 | 1 | 1 | 1 | 1 | 1 | 1 | 1 | 1 | NA | 1 | NA |
| Armangue T, Spatola M, Vlagea A, et al. Frequency, symptoms, risk factors, and outcomes of autoimmune encephalitis after herpes simplex encephalitis: a prospective observational study and retrospective analysis. Lancet Neurol. 2018;17(9):760-772. doi:10.1016/S1474-4422(18)30244-8 | 62 | 2018 | 1 | 1 | 1 | 1 | 1 | 1 | 1 | 1 | 1 | 1 | 1 | 0 |
| Zhang J, Liu J, Wang J, Gan J. Anti-NMDAR encephalitis secondary to acute necrotizing encephalopathy caused by herpes simplex virus infection in infants: Case series. Clin Neurol Neurosurg. 2023;233:107955. doi:10.1016/j.clineuro.2023.107955 | 4 | 2023 | 1 | 1 | 1 | Unclear | 1 | 1 | 1 | 1 | 0 | NA | 1 | NA |
| Quade A, Rostasy K, Wickström R, et al. Autoimmune Encephalitis with Autoantibodies to NMDAR1 following Herpes Encephalitis in Children and Adolescents. Neuropediatrics. 2023;54(1):14-19. doi:10.1055/s-0042-1757706 | 15 | 2002 | 1 | 1 | 1 | Unclear | 1 | 1 | 1 | 1 | 0 | 1 | 1 | 1 |
| Klein da Costa B, de Oliveira Pinto P, Staub L, et al. Neurological syndromes and potential triggers associated with antibodies to neuronal surface antigens. Mult Scler Relat Disord. 2023;80:105022. | 3 | 2023 | 1 | 1 | 1 | Unclear | 1 | 1 | 1 | 1 | 0 | 1 | 1 | 0 |
| Carrascosa-Garcia P, Oviedo-Melgares L, Torres-Fernandez D, et al. Rituximab as second-line treatment in anti-NMDAR encephalitis after herpes simplex encephalitis in children. Indian J Pediatr. 2022;89(10):1031-1033. | 2 | 2022 | 1 | 1 | 1 | Unclear | 1 | 1 | 1 | 1 | 0 | 1 | 1 | 0 |
| Dorcet G, Benaiteau M, Bost C, et al. Two cases of late-onset anti-NMDAr auto-immune encephalitis after herpes simplex virus 1 encephalitis. Front Neurol. 2020;11:38. | 2 | 2020 | 1 | 1 | 1 | Unclear | 1 | 1 | 1 | 1 | 0 | NA | 1 | NA |
| Alexopoulos H, Akrivou S, Mastroyanni S, et al. Postherpes simplex encephalitis: a case series of viral-triggered autoimmunity, synaptic autoantibodies and response to therapy. Ther Adv Neurol Disord. 2018;11:1756286418768778. | 5 | 2018 | 1 | 1 | 1 | Unclear | 1 | 1 | 1 | 1 | 0 |  | 1 | NA |
| Brás A, André A, Sá L, et al. Anti-NMDAR encephalitis following herpes simplex virus encephalitis: 2 cases from Portugal. Neurohospitalist. 2020;10(2):133-138. | 2 | 2020 | 1 | 1 | 1 | Unclear | 1 | 1 | 1 | 1 | 1 |  | 1 | NA |
| Marcus L, Ness JM. Pediatric N-methyl-d-aspartate (NMDA) receptor encephalitis, with and without herpes encephalitis. J Child Neurol. 2021;36(9):743-751. | 6 | 2021 | 1 | 1 | 1 | 1 | 1 | 1 | 1 | 1 | 0 | 1 | 1 | NA |
| Armangue T, Moris G, Cantarín-Extremera V, et al. Autoimmune post-herpes simplex encephalitis of adults and teenagers. Neurology. 2015;85(20):1736-1743. | 8 | 2015 | 1 | 1 | 1 | 1 | 1 | 1 | 1 | 1 | 1 | 1 | 1 | NA |
| Sandweiss AJ, Erickson TA, Jiang Y, et al. Infectious profiles in pediatric anti-N-methyl-D-aspartate receptor encephalitis. J Neuroimmunol. 2023;381:578139. | 11 | 2023 | 1 | 1 | 1 | Unclear | 1 | 1 | 1 | 1 | 1 | 1 | 1 | 1 |
| Dumez P, Villagrán-García M, Bani-Sadr A, et al. Specific clinical and radiological characteristics of anti-NMDA receptor autoimmune encephalitis following herpes encephalitis. J Neurol. Published online August 16, 2024. doi:10.1007/s00415-024-12615-7. | 13 | 2024 | 1 | 1 | 1 | Unclear | 1 | 1 | 1 | 1 | 1 | 1 | 1 | 1 |
| Swayne A, Warren N, Prain K, Gillis D, Wong R, Blum S. Analysing triggers for anti-NMDA-receptor encephalitis including herpes simplex virus encephalitis and ovarian teratoma: results from the Queensland Autoimmune Encephalitis cohort. Intern Med J. 2022;52(11):1943-1949. doi:10.1111/imj.15472. | 3 | 2022 | 1 | 1 | 1 | Unclear | 1 | 1 | 1 | 1 | 0 | NA | 1 | 1 |
| Søgaard A, Poulsen CA, Belhouche NZ, et al. Post-herpetic anti-NMDAR encephalitis in Denmark: current status and future challenges. Biomedicines. 2024;12(9):1953. doi:10.3390/biomedicines12091953. | 8 | 2024 | 1 | 1 | 1 | Unclear | 1 | 1 | 1 | 1 | 1 | 1 | 1 | 1 |
| Quade A, Rostasy K, Wickström R, Aydin ÖF, Sartori S, Nosadini M, Knierim E, Kluger G, Korinthenberg R, Stüve B, Waltz S, Leiz S, Häusler M. Autoimmune Encephalitis with Autoantibodies to NMDAR1 following Herpes Encephalitis in Children and Adolescents. Neuropediatrics. 2023 Feb;54(1):14-19. doi: 10.1055/s-0042-1757706. Epub 2022 Dec 21. PMID: 36543183. | 4 | 2023 | 1 | 1 | 1 | Unclear | 1 | 1 | 1 | 1 | 0 | 1 | 1 | 1 |

**References**

1. Graus F, Titulaer MJ, Balu R, et al. A clinical approach to diagnosis of autoimmune encephalitis. *Lancet Neurol*. 2016;15(4):391. doi:10.1016/S1474-4422(15)00401-9

2. Bigi S, Fischer U, Wehrli E, et al. Acute ischemic stroke in children versus young adults. *Ann Neurol*. 2011;70(2):245-254. doi:10.1002/ANA.22427

3. Al-Diwani A, Handel A, Townsend L, et al. The psychopathology of NMDAR-antibody encephalitis in adults: a systematic review and phenotypic analysis of individual patient data. *Lancet Psychiatry*. 2019;6(3):235-246. doi:10.1016/S2215-0366(19)30001-X

4. Armangue T, Spatola M, Vlagea A, et al. Frequency, symptoms, risk factors, and outcomes of autoimmune encephalitis after herpes simplex encephalitis: a prospective observational study and retrospective analysis. *Lancet Neurol*. 2018;17(9):760-772. doi:10.1016/S1474-4422(18)30244-8

5. Munn Z, Barker TH, Moola S, et al. Methodological quality of case series studies: an introduction to the JBI critical appraisal tool. *JBI Evid Synth*. 2020;18(10). doi:10.11124/JBISRIR-D-19-00099

6. Nosadini M, Eyre M, Molteni E, et al. Use and Safety of Immunotherapeutic Management of N-Methyl-d-Aspartate Receptor Antibody Encephalitis: A Meta-analysis. *JAMA Neurol*. 2021;78(11):1333-1344. doi:10.1001/JAMANEUROL.2021.3188
