# Supplementary material for: Clinical phenotype and outcomes in autoimmune encephalitis after herpes simplex virus encephalitis: A systematic review and meta-analysis
Source: J Infect. Author manuscript; Available in PMC 2026 Jan 29. (PMC7618681; doi:10.1016/j.jinf.2025.106566)
Supplement: Supplementary Material [file EMS212239-supplement-Supplementary_Material.zip › 1-s2.0-S0163445325001604-mmc4.pdf]

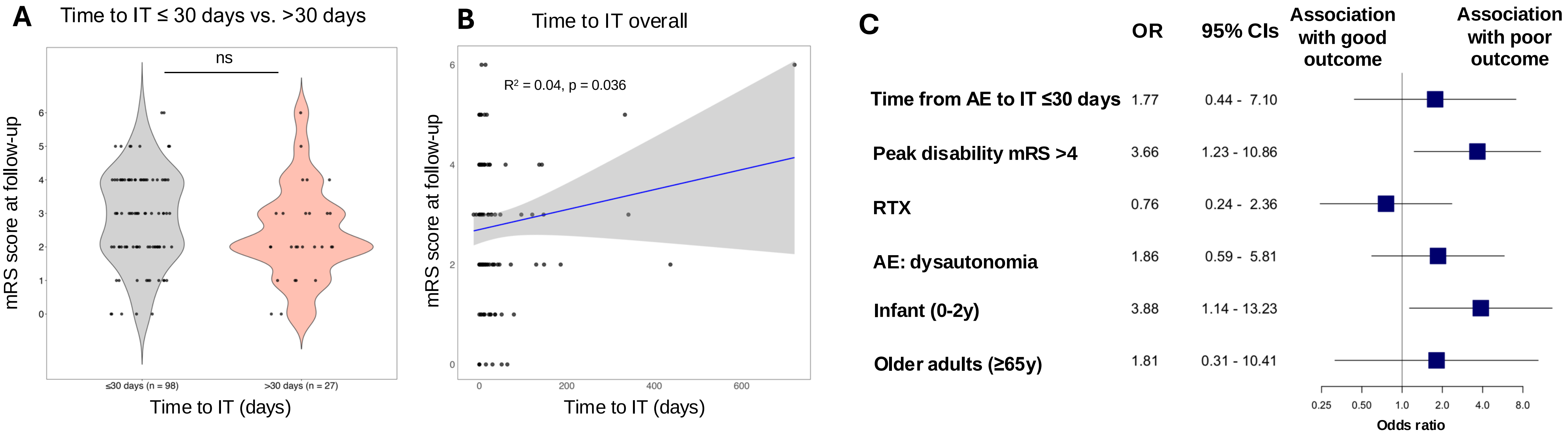
A
Time to IT ≤ 30 days vs. >30 days
ns
mRS score at follow-up
0
1
2
3
4
5
6
≤30 days (n = 98)
>30 days (n = 27)
Time to IT (days)
B
Time to IT overall
R^2 = 0.04, p = 0.036
mRS score at follow-up
0
2
4
6
0
200
400
600
Time to IT (days)
C
OR
95% CIs
Association with good outcome
Association with poor outcome
Time from AE to IT ≤30 days
1.77
0.44 - 7.10
Peak disability mRS >4
3.66
1.23 - 10.86
RTX
0.76
0.24 - 2.36
AE: dysautonomia
1.86
0.59 - 5.81
Infant (0-2y)
3.88
1.14 - 13.23
Older adults (≥65y)
1.81
0.31 - 10.41
0.25
0.50
1.0
2.0
4.0
8.0
Odds ratio
