# Supplementary material for: Clinical phenotype and outcomes in autoimmune encephalitis after herpes simplex virus encephalitis: A systematic review and meta-analysis
Source: J Infect. Author manuscript; Available in PMC 2026 Jan 29. (PMC7618681; doi:10.1016/j.jinf.2025.106566)
Supplement: Supplementary Material [file EMS212239-supplement-Supplementary_Material.zip › 1-s2.0-S0163445325001604-mmc2.pdf]

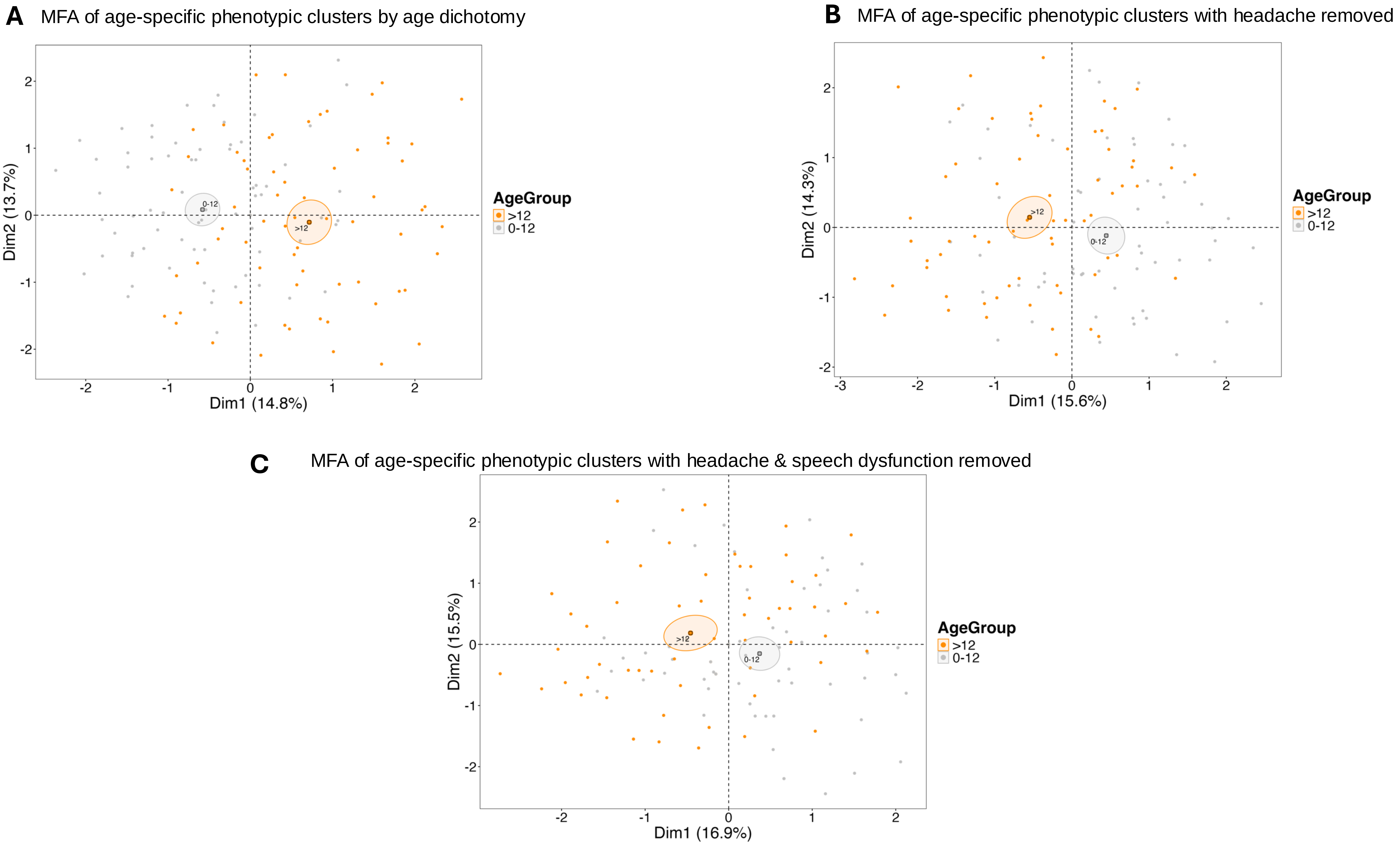
A
MFA of age-specific phenotypic clusters by age dichotomy
Dim1 (14.8%)
Dim2 (13.7%)
AgeGroup
>12
0-12
B
MFA of age-specific phenotypic clusters with headache removed
Dim1 (15.6%)
Dim2 (14.3%)
AgeGroup
>12
0-12
C
MFA of age-specific phenotypic clusters with headache & speech dysfunction removed
Dim1 (16.9%)
Dim2 (15.5%)
AgeGroup
>12
0-12
