# Supplementary material for: Clinical phenotype and outcomes in autoimmune encephalitis after herpes simplex virus encephalitis: A systematic review and meta-analysis
Source: J Infect. Author manuscript; Available in PMC 2026 Jan 29. (PMC7618681; doi:10.1016/j.jinf.2025.106566)

Regression model excluding Armangue et al 2018 without imputation

A

Characteristic	OR	95% CIs	P value
AE: neuropsychiatric	3.31	1.19 - 9.25	.022
RTX	0.43	0.19 - 0.99	.046
Peak disability mRS >4	2.68	1.21 - 5.94	.015
Infant (0-2y)	11.00	4.25 - 28.44	<0.0001
Older adults (≥65y)	4.27	1.22 - 14.88	.022

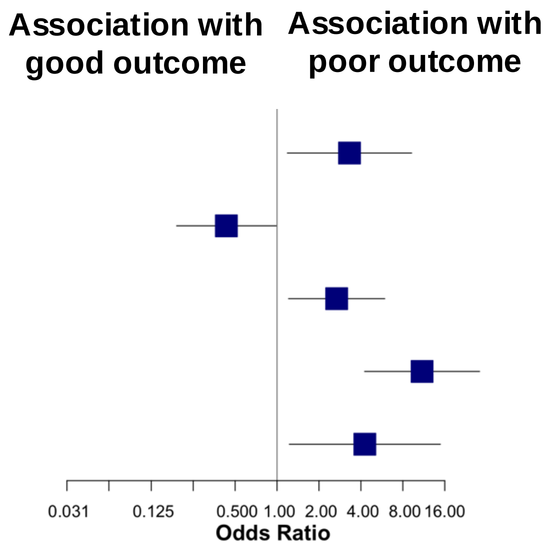

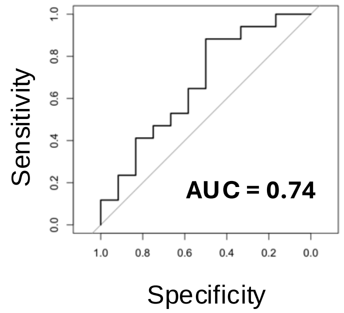

Regression model including Armangue et al 2018 with 10% imputation

B

Characteristic	OR	95% CIs	P value
AE: neuropsychiatric	3.65	1.55 - 8.58	.003
RTX	0.57	0.28 - 1.16	.120
Peak disability mRS >4	2.03	1.05 - 3.91	.035
Infant (0-2y)	14.00	5.88 - 33.37	<0.0001
Child (3-12y)	2.62	1.04 - 6.59	.041
Older adults (≥65y)	7.50	2.46 - 22.83	.0004

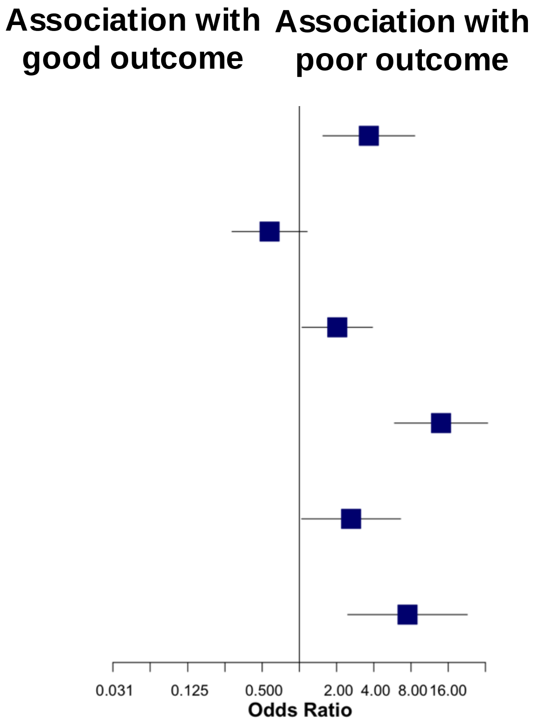

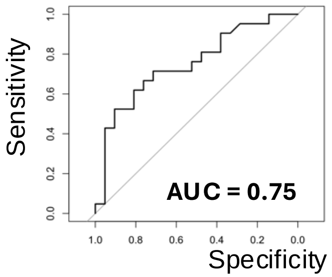

Supplement: Supplementary Material [file EMS212239-supplement-Supplementary_Material.zip › 1-s2.0-S0163445325001604-mmc3.pdf]
